# Health of Aboriginal and Torres Strait Islander children and their grandparents: a Western Australian retrospective cohort study

**DOI:** 10.1186/s12889-025-24577-0

**Published:** 2025-10-09

**Authors:** Alison Gibberd, Jocelyn Jones, Louisa Jorm, Carrington Shepherd, Sandra Eades, Bridgette McNamara

**Affiliations:** 1 Melbourne School of Population and Global Health, The University of Melbourne, Melbourne, VIC 3010 Australia; 2 National Drug Research Institute, Curtin University, Kent Street, Bentley, Perth, WA 6102 Australia; 3 Centre for Big Data Research in Health, The University of New South Wales, High St, Kensington, Sydney, NSW 2052 Australia; 4 Curtin Medical School, Curtin University, Kent Street, Bentley, Perth, WA 6102 Australia; 5 Ngangk Yira Institute, Murdoch University, 90 South Street, Murdoch, Perth, WA 6150 Australia

**Keywords:** Aboriginal and torres strait islander people, Indigenous peoples, Grandparents, Child health, Linked data

## Abstract

**Background:**

In Australian Aboriginal and Torres Strait Islander communities, childcare is traditionally shared by kin. Little is known about how grandparental care impacts Aboriginal child health and evidence from other countries is mixed. We explored relationships between grandparental health (a proxy for grandparental care) and health and health service use by Aboriginal children born in Western Australia from 2000 to 2013.

**Methods:**

This is a retrospective cohort study using linked administrative health data. Outcomes were child mortality, hospital admissions, and emergency department (ED) presentations up to five years old. Grandparental health when the child was born was categorised as healthy (none/one Elixhauser condition), unhealthy (two or more conditions), or deceased. Grandparental-child health associations were estimated using regression with adjustment for birth year, sex, remoteness, socioeconomic advantage, maternal smoking, and maternal age.

**Results:**

29,409 Aboriginal children linked to their maternal grandmothers. 70% also linked to maternal grandfathers, 66% to paternal grandmothers, and 49% to paternal grandfathers. 86% of maternal grandmothers were healthy, 7% unhealthy, and 7% deceased. Children with healthy grandmothers had an average of 27% fewer hospital days (adjusted incidence rate ratio: 0.73, 95% CI: 0.65, 0.83) than those with deceased grandmothers. They also had lower mortality and fewer potentially avoidable admissions and emergency department presentations. Children with unhealthy or deceased grandmothers had comparable rates. These patterns were similar for all four grandparents, but generally stronger for maternal grandmothers. Stillbirth and unavoidable admissions were unrelated to grandparental health.

**Conclusions:**

Aboriginal children with healthy grandparents had better health and lower health service use. If this relationship is causal, healthy ageing and strong family connections must be supported to improve child health. Even if the relationship is not causal, healthy ageing, a family-centred approach to health care, and social support may help families experiencing poor health in multiple generations simultaneously.

**Supplementary Information:**

The online version contains supplementary material available at 10.1186/s12889-025-24577-0.

## Background

Children have a special place within Australian Aboriginal and Torres Strait Islander families and communities and traditionally child-rearing responsibilities are shared within kinship systems [1]. While Aboriginal and Torres Strait Islander (hereafter referred to as Aboriginal) communities across the country differ culturally, some elements are shared. From a young age, children are taught who their relatives are and are comfortable with them and, as children grow older, relatives become more responsible for caring for them [1]. In addition to ‘hands-on’ care, kin may have special obligations. For grandparents, this may include imparting cultural knowledge and strengthening the child’s ties with the rest of the extended family.

Colonisation has caused significant social disruption; many Aboriginal families have been fractured by early mortality, incarceration, and the forced removal of children over multiple generations [2, 3]. Despite this, kin continue to play an important role in communities, particularly grandparents. For example, a study of Martu families from a very remote region in the state of Western Australia (WA) found that, after mothers, grandmothers spent the most time caring for children [4] and when children are removed from their parents and placed in out-of-home care, grandparents are the most likely relatives to become their primary carers [5]. 

At a population level, it is challenging to accurately describe the nature of the relationships between children and their grandparents. National surveys have, to some degree, quantified how many Aboriginal children are cared for by their grandparents, from full-time to occasional care. When the Longitudinal Study of Indigenous Children cohort was largely under 5 years, a grandparent was the primary caregiver for 3% of children, 6% lived in the same house as a grandparent, and 49% of children were cared for by grandparents when their primary caregiver was not available [6, 7]. In the Longitudinal Study of Australian Children, 12% of Aboriginal children aged 0 or 1 year lived in the same household as a grandmother and 7% in the same household as a grandfather, compared to 6% and 4%, respectively, for non-Aboriginal children [8]. Aboriginal children were also more likely to live in the same house as aunts, uncles, and other relatives [9]. While these surveys, they do not fully capture the complexity of relationships between children and their grandparents, they suggest that around one in ten young Aboriginal children live in the same household as a grandparent and around half receive care from grandparents when their parents are unavailable. However, the frequency of care and the level of contact between grandparents and the remaining half of children is unknown.

A number of quantitative studies have examined associations between grandparental care, co-habitation, and survival [10] and child health and development outcomes among populations from high- to low-income countries, with mixed results [10]. Among children raised in the custody of their grandparents, health was poorer, on average, than children raised by their parents – possibly because such families are among the most socioeconomically deprived [10]. However, in situations of multigenerational care, when care is given by both parents and grandparents, the results were more inconsistent. Some studies found positive effects on child height, weight, and injury, while others found that children who lived with grandparents had higher body mass index, delayed physical development, and reduced physical function [10]. 

Studies have found some evidence of increased child survival for children with living grandmothers. Sear and Coall (2010) found that among eleven studies that attempted to address confounding, seven (64%) reported a positive association between child survival and having a living maternal grandmother, one reported a negative association and three found no association [11]. A further two studies that did not address confounding found a positive association between child and maternal grandmaternal survival. The populations were all pre-industrial (for example, central Japan from 1671 to 1871 [12], a parish in Poland from 1737 to 1768 [13], and the Krummhörn region in Germany from 1720 to 1874^14^) or in low- or middle-income nations with high rates of child mortality (for example, rural Gambia from 1950 to 1974^15^ and Khasi families in India from 1980 to 2000^11^). Results were similar for studies including paternal grandmothers, but most studies showed no association or a negative one for grandfathers [11]. 

To the best of our knowledge, no such studies have been undertaken among Aboriginal Australian children, who have poorer health than non-Aboriginal children across a wide range of measures, including gestational age, birthweight, infections in childhood, asthma, and injury [16–19]. Those environments where Aboriginal children flourish must be identified and supported. Involved grandparents are acknowledged as an important component of the social and emotional wellbeing of children and families, but may also contribute to better physical wellbeing among children.

Given the inconsistent results from research outside Australia, the lack of studies in Australia, the urgent need to improve Aboriginal child health, and the importance of grandparents in Aboriginal communities, we explored whether having healthy grandparents was associated with the health and health service use of young Aboriginal children born in Western Australia from 2000 to 2013 using linked administrative health data.

## Methods

### Data sources

Records from the Midwives Notification System (birth record), birth registrations, Hospital Morbidity Data Collection, Emergency Department Data Collection, Mental Health Information System, child protection data, and death registrations were linked probabilistically by Western Australia Data Linkage Services [20]. This study was part of the larger Defying the Odds project, an Aboriginal-led study of Aboriginal child health.

### Study sample

The study sample was all Aboriginal children born from 2000 to 2013 and recorded in WA’s Midwives Notification System, who linked to their maternal grandmothers. Aboriginal status was identified according to an Aboriginal and/or Torres Strait Islander Flag’ in the WA Data Linkage System. The indicator is based on an algorithm applied to a range of administrative data sources [21]. The algorithm strikes a balance between failing to identify Aboriginal people and incorrectly categorising a non-Aboriginal person as Aboriginal [21, 22]. The study sample included (1) children recorded as Aboriginal unless a full sibling was categorised as non-Aboriginal and (2) children whose parents or grandparents were recorded as Aboriginal.

Western Australia Data Linkage Services linked children to their grandparents by first linking the child to their parents, and then linking their parents to their grandparents. These links are possible using birth registrations, which list mothers and fathers, and the Midwives Notifications System, which lists mothers. Children could not be linked to a grandparent if: (1) a parent was born outside WA; (2) birth registrations of the child or a parent did not record the father’s name, preventing linkage through a paternal line; [23] (3) the birth of the child or birth of a parent was not registered; [24] or (4) linkage failure.

### Outcomes

Mortality outcomes were stillbirth from 20 full weeks’ gestation onwards and death within five years of a live birth. Health service utilisation in the first five years of life was: (1) total hospital bed days excluding the birth admission; (2) the number of potentially avoidable hospital admissions, excluding injury; (3) any potentially avoidable hospital admission for the eight most common diagnoses (dental, asthma, pneumonia, gastroenteritis, otitis media, skin infection, acute bronchiolitis, and viral infection of unspecified site); (4) any admission for unintentional injury; (5) any unavoidable admissions; and (6) number of emergency department (ED) presentations. Multiple ED presentations in one day were only counted once. ED data were not available for 2000 or 2001 so analysis was restricted to 2002 onwards.

Potentially avoidable hospital admissions are admissions that may not have occurred if families had adequate socioeconomic resources, a safe physical environment, access to appropriate primary healthcare, and other reasons [25]. Anderson et al. developed consensus lists of International Statistical Classification of Diseases and Related Health Problems, Tenth Revision, Australian Modification (ICD-10-AM) diagnosis codes to identify potentially avoidable admissions, as well as unavoidable admissions (for example, for cystic fibrosis). We used these lists and the primary diagnosis codes recorded in hospital admissions to identify potentially avoidable admissions. Admissions in the first 28 days of life and planned admissions (except planned dental admissions) were excluded.

Children were categorised as having an admission for an unintentional injury if the principal diagnosis was injury (ICD-10-AM codes S00-T75 or T79) and the first external code indicated the injury was unintentional (V01-X59 or Y85-Y86).

### Study factors

Grandparental health when the child was born was classified as ‘healthy’ or ‘unhealthy’, based on the Elixhauser comorbidities [26], or ‘deceased’ (determined from death registrations). The thirty-one Elixhauser comorbidities include conditions such as diabetes, hypertension, renal disease, alcohol misuse, and depression and the number of conditions is predictive of a range of poor outcomes, including longer hospital stay, post-operative complications, and mortality [27]. The unweighted Elixhauser score was used (the number of conditions) and included diagnoses [28] recorded in hospital admissions in the year before the child’s birth. A lookback period of one year can improve prediction of mortality [29]. In addition to this classification of healthy and unhealthy, we also examined whether there was a ‘dose-response’ trend between child health and Elixhauser scores of 0, 1, 2, and 3 or more.

### Other factors

We also report the child’s year of birth, sex, maternal age when the child was born, maternal smoking during pregnancy, grandparental age when the child was born, socioeconomic advantage, and remoteness. Area-level socioeconomic advantage was based on the Indigenous Relative Socioeconomic Outcomes (IRSEO) Index [30]. The child’s Statistical Area 1 (SA1) at birth was mapped to their Indigenous Region using ABS geographical correspondence files, which was then mapped to an IRSEO ranking [31]. The children’s IRSEO rankings were then aggregated to form socioeconomic advantage quartiles. The remoteness of the child’s SA1 at birth was based on 2011 ARIA+ (Accessibility/Remoteness Index for Australia) categories (major cities, inner regional, outer regional, remote, and very remote) [32]. 

### Analysis

Associations between the health of each of the four grandparents and the health of the grandchildren were estimated using regression models. Odds ratios (ORs) were estimated using logistic regression for the binary outcomes. Incidence rate ratios (IRRs) were estimated using negative binomial models for count outcomes, accounting for time at risk. Time at risk did not include the infant’s hospital stay for their birth admission. For the number of potentially avoidable hospital admissions and ED presentations, time at risk also excluded any subsequent time in hospital, barring the initial day of each admission. For all models, a generalised estimating equation approach, with an exchangeable correlation matrix, accounted for clustering by mother.

**Fig. 1:**
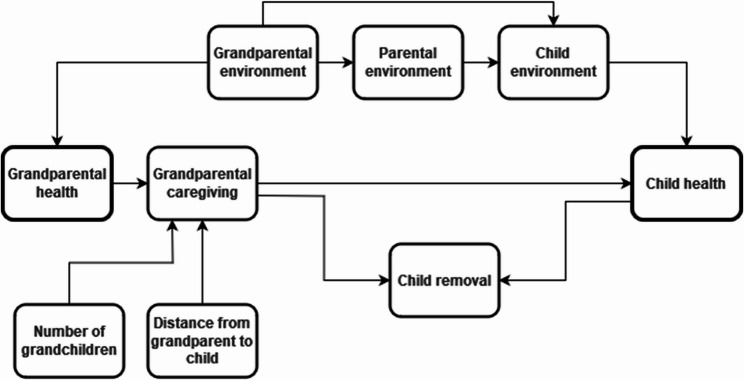
Directed acyclic graph of causal relationships between grandparental health and child health

Minimal models adjusted for child’s year of birth and sex only. A directed acyclic graph (Fig. 1) indicated that environmental factors shared by children and their grandparents might confound the grandparental-child health association and adjustment for either the child’s environment or the grandparent’s environment was needed. Therefore, the fully adjusted models also included components of the child’s environment – remoteness, area-level socioeconomic advantage, and indicators of individual-level socioeconomic advantage (maternal age when the child was born and maternal smoking during pregnancy). Maternal smoking may also be a proxy for maternal grandparental smoking, a risk factor for grandparental health, and exposure to smoke in early childhood, a health risk for the cohort. The removal of children from their parents by child protection authorities was not considered a confounder but was included in the figure to provide context for the sensitivity analysis which excluded children subject to removal before the age of five.

Unavoidable hospital admissions were chosen as a negative control, as we did not expect grandparental health to affect their grandchildren’s unavoidable hospital admissions. We hypothesised that if a relationship was found, it may result from both grandparents and children accessing hospitals with high propensities to admit patients (for example, hospitals in areas with poor access to quality and timely primary care). A high propensity to admit could simultaneously (1) increase the likelihood of grandparents being categorised as unhealthy in this study and (2) increase child hospital admissions and ED presentations. However, if no relationship was found for unavoidable hospital admissions, propensity to admit may not be relevant to this study.

E-values were calculated for the fully adjusted ORs and IRRs [33, 34]. E-values indicate the unmeasured confounding required for an estimate to equal one. The greater the E-value, the greater the residual confounding required to fully explain the observed association, and the greater the plausibility of a causal relationship.

Models that included the health of all four grandparents simultaneously were also fit for the subset of children who linked to all grandparents. Estimates were compared to see if associations were stronger for maternal grandmothers who are often the most active caregivers.

To assess whether results varied for Aboriginal and non-Aboriginal maternal grandmothers, we also fit models that included an interaction between grandmaternal health and Indigeneity (as recorded by her Aboriginal and/or Torres Strait Islander Flag’), after excluding those with unknown Indigeneity. Models with interactions between grandparental health and remoteness, socioeconomic advantage, and the number of grandchildren born before the child’s fifth birthday (that is, the number of siblings and cousins sharing that grandparent by age 5) were also fit. We also examined whether associations were different for grandparent-child pairs who lived close to each other (defined as in the same Indigenous Region) than those living in different regions. Maternal grandmothers’ Indigenous Region was determined by mapping SA1s, recorded in her hospital admissions, ED presentations, mental health records, or death records, in the first five years of the child’s life. The record closest to the grandchild’s date of birth was selected and this analysis only included grandmothers who were alive when the child was born and had at least one relevant health record in the first five years of the child’s life.

A sensitivity analysis excluding children who entered formal out-of-home care (placements outside the family home which are managed by the state) at least once in the first five years of life was undertaken. These children may have had limited contact with grandparents after removal or a grandparent may have become their primary caregiver. A second sensitivity analysis restricted the analysis to those whose grandparents had at least one hospital, ED, or mental health record in the ten years before the birth of the child. The rationale was that some grandparents may have been misclassified as ‘healthy’ because of an absence of records, potentially because they had left WA, or failed to link to their hospital records.

Stillbirth and death before age five were rare and not included in further analysis after the initial main analysis. Unavoidable admissions were also not examined further as they were a negative control with no evidence of association with grandparental health in the main analysis.

SAS software, Version 9.4 of the SAS System for Windows was used for the regression analysis and R 4.2.1 was used to calculate the E-values [35]. 

## Results

### Study sample

**Fig. 2:**
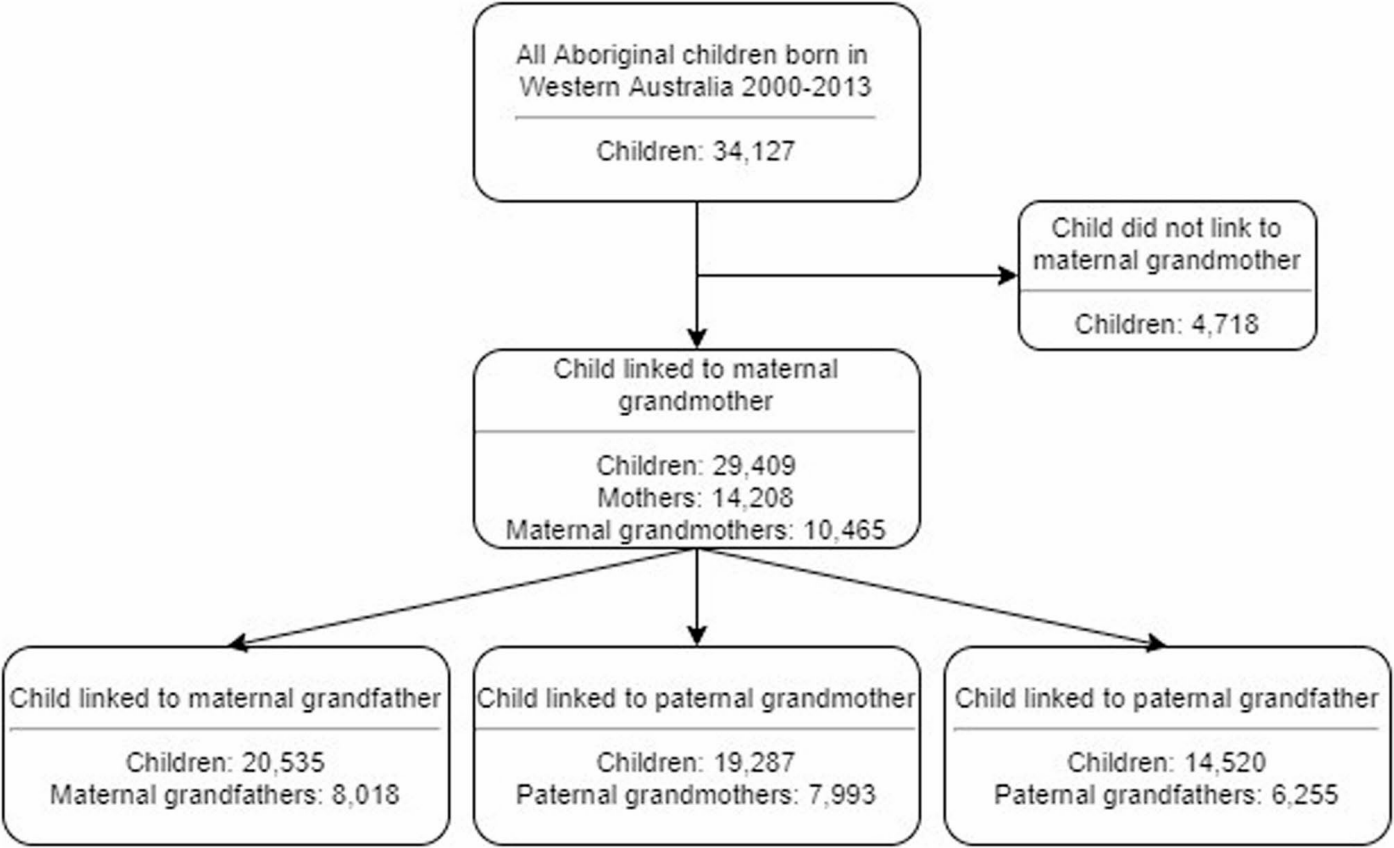
Selection of study sample

The number of Aboriginal children born in WA from 2000 to 2013 was 34,127. The study sample comprised the 29,409 (86%) children who linked to 10,465 maternal grandmothers (Fig. 2). Maternal grandmothers were Aboriginal for 72% of the sample (21,248 children), non-Aboriginal for 24% (7181 children), and unknown status for 3% (980 children). The mothers of the children who could not be linked did not have a WA birth record or birth registration. Of the study sample, 20,535 (70%) linked to their maternal grandfather (Aboriginal for 59% [12,110 children], non-Aboriginal for 36% [7346 children], and unknown status for 5% [1079 children]), 19,287 (66%) to their paternal grandmother (Aboriginal for 71% [13,749 children], non-Aboriginal for 24% [4579 children], and unknown status for 5% [959 children]), and 14,520 (49%) to their paternal grandfather (Aboriginal for 58% [8434 children], non-Aboriginal for 35% [5019 children], and unknown status for 7% [1067 children]).

Children who linked to their maternal grandfather or paternal grandparents were from more advantaged and less remote areas than those without links (Table 1). For example, 45% of those who linked to their maternal grandfather were from major cities, compared to 31% of those who could not be linked. A majority of maternal grandmothers were less than 50 years old when their grandchild was born (58%), compared to 41% of maternal grandfathers, 48% of paternal grandmothers, and 32% of paternal grandfathers, among the children with links.

**Table 1:** Characteristics of children born in WA 2000–2013 who linked to their maternal grandmother by whether they were linked to their other 3 grandparents

Characteristic	Maternal grandmother	Maternal grandfather		Paternal grandmother		Paternal grandfather	
	Linked *n* (%)	Linked *n* (%)	Unlinked *n* (%)	Linked *n* (%)	Unlinked *n* (%)	Linked *n* (%)	Unlinked *n* (%)
Total	29,409 (100)	20,535 (70)	8874 (30)	19,287 (66)	10,122 (34)	14,520 (49)	14,889 (51)
Infant year of birth							
2000–2004	7591 (26)	5167 (25)	2424 (27)	4887 (25)	2704 (27)	3643 (25)	3948 (27)
2005–2009	10,588 (36)	7312 (36)	3276 (37)	6853 (36)	3735 (37)	5175 (36)	5413 (36)
2010–2013	11,230 (38)	8056 (39)	3174 (36)	7547 (39)	3683 (36)	5702 (39)	5528 (37)
Infant sex							
Male	14,913 (51)	10,397 (51)	4516 (51)	9817 (51)	5096 (50)	7382 (51)	7531 (51)
Female	14,494 (49)	10,136 (49)	4358 (49)	9470 (49)	5024 (50)	7138 (49)	7356 (49)
Maternal age							
< 20	6373 (22)	4259 (21)	2114 (24)	4103 (21)	2270 (22)	2881 (20)	3492 (23)
20–24	9564 (33)	6584 (32)	2980 (34)	6400 (33)	3164 (31)	4783 (33)	4781 (32)
25–29	7225 (25)	5021 (24)	2204 (25)	4766 (25)	2459 (24)	3640 (25)	3585 (24)
30–34	4221 (14)	3134 (15)	1087 (12)	2758 (14)	1463 (14)	2222 (15)	1999 (13)
35+	2026 (7)	1537 (7)	489 (6)	1260 (7)	766 (8)	994 (7)	1032 (7)
Area-level socioeconomic advantage							
Quartile 1 (most advantaged)	6794 (23)	5309 (26)	1485 (17)	4647 (24)	2147 (21)	3781 (26)	3013 (20)
Quartile 2	7447 (25)	5527 (27)	1920 (22)	5152 (27)	2295 (23)	3994 (28)	3453 (23)
Quartile 3	6792 (23)	4948 (24)	1844 (21)	4624 (24)	2168 (21)	3605 (25)	3187 (21)
Quartile 4	8345 (28)	4734 (23)	3611 (41)	4852 (25)	3493 (35)	3139 (22)	5206 (35)
Maternal remoteness of residence							
Major cities	12,041 (41)	9318 (45)	2723 (31)	8304 (43)	3737 (37)	6654 (46)	5387 (36)
Inner regional	1859 (6)	1545 (8)	314 (4)	1314 (7)	545 (5)	1145 (8)	714 (5)
Outer regional	4782 (16)	3525 (17)	1257 (14)	3293 (17)	1489 (15)	2567 (18)	2215 (15)
Remote	5565 (19)	3582 (17)	1983 (22)	3519 (18)	2046 (20)	2500 (17)	3065 (21)
Very remote	5131 (17)	2548 (12)	2583 (29)	2845 (15)	2286 (23)	1653 (11)	3478 (23)
Maternal smoking during pregnancy							
No	16,023 (54)	11,966 (58)	4057 (46)	11,185 (58)	4838 (48)	8774 (60)	7249 (49)
Yes	13,386 (46)	8569 (42)	4817 (54)	8102 (42)	5284 (52)	5746 (40)	7640 (51)
Grandparental age							
< 50	17,145 (58)	8371 (41)	-	9352 (48)	-	4712 (32)	-
50–59	8763 (30)	7366 (36)	-	6395 (33)	-	5218 (36)	-
60–69	2203 (7)	2921 (14)	-	2040 (11)	-	2560 (18)	-
70+	318 (1)	795 (4)	-	561 (3)	-	972 (7)	-
Unknown	980 (3)	1082 (5)	8874 (100)	939 (5)	10,122 (100)	1058 (7)	14,889 (100)
Infant sex was unknown for 2 infants, socioeconomic disadvantage and remoteness were unknown for 31 infants, and grandparental age was unknown for 980 maternal grandmothers, 9,956 maternal grandfathers, 11,061 paternal grandmothers, and 15,947 paternal grandfathers. The main reason for missing grandparental age was failure to link the infant and the grandparent. Percents are column percents, except the ‘Total’ row which is a row percent							

### Grandparental health

The vast majority (81–86%) of grandparents were categorised as healthy when the child was born (Table 2). Grandmothers were more likely to be alive (for example, 93% of maternal grandmothers versus 87% of maternal grandfathers).

**Table 2:** Health of grandparents at time of birth of Aboriginal children born in WA 2000–2013

		Healthy *n* (%)	Unhealthy *n* (%)	Dead *n* (%)
Maternal	Grandmother	25,151 (86)	2066 (7)	2192 (7)
	Grandfather	16,779 (82)	1079 (5)	2677 (13)
Paternal	Grandmother	16,458 (85)	1289 (7)	1540 (8)
	Grandfather	11,703 (81)	753 (5)	2064 (14)

Grandparents with an unweighted Elixhauser score of 0 or 1 were classified as ‘healthy’ and those with a score of 2 or more as ‘unhealthy’

### Child health and service use

**Fig. 3:**
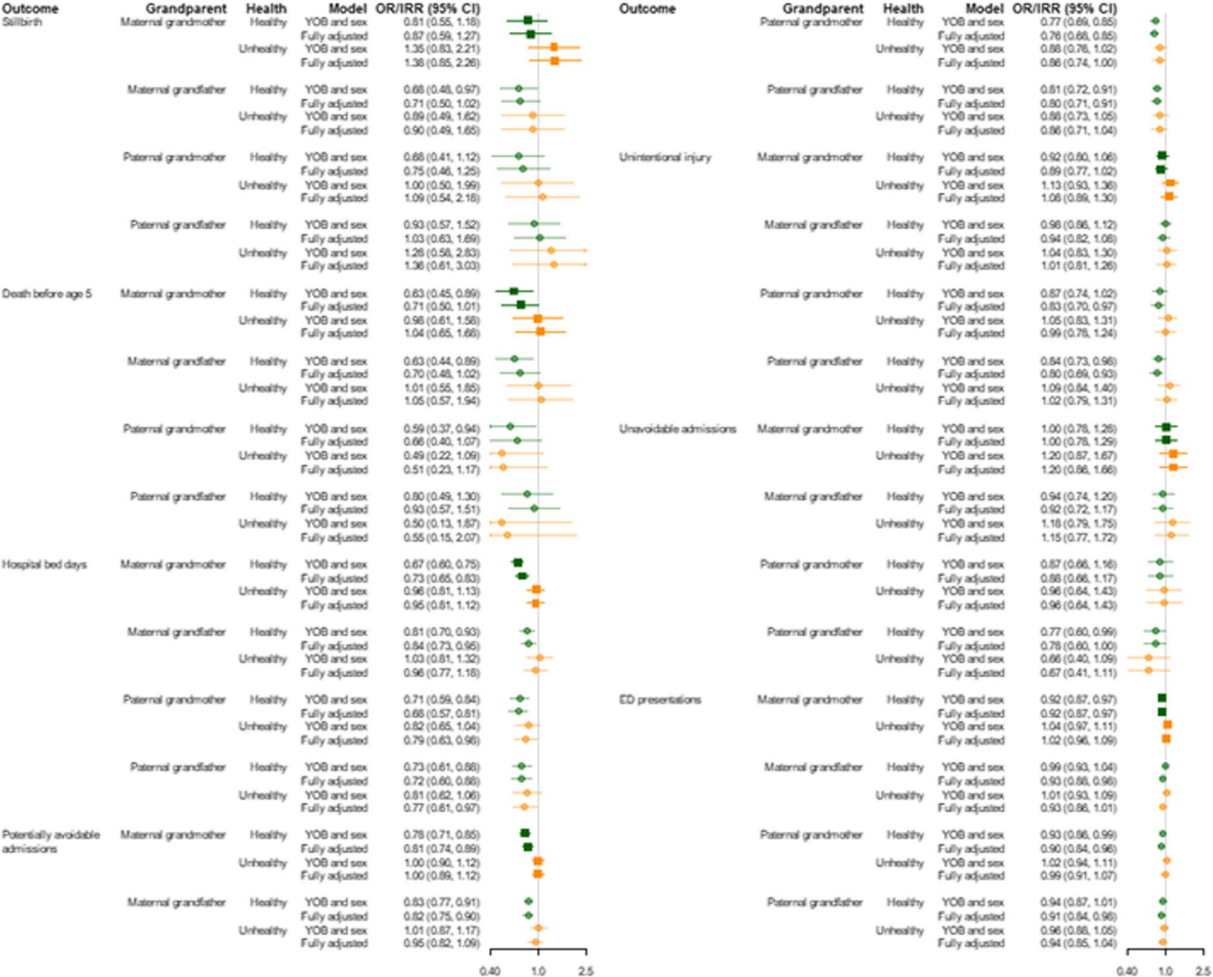
Associations (odds ratios and incidence rate ratios) between child health outcomes and health service use and grandparental health Models adjusted for the child’s year of birth (YOB) and sex or fully adjusted (child’s year of birth, sex, maternal age, maternal smoking during pregnancy, socioeconomic advantage, and remoteness). OR: Odds ratio (stillbirth, death before age 5, any admission for unintentional injury, any unavoidable admission). IRR: Incidence rate ratio (number of hospital bed days, potentially avoidable admissions, emergency department (ED) admissions). CI: Confidence interval

Stillbirth was less common among children with healthy grandparents than those with unhealthy or deceased grandparents (Table 3; Fig. 3 and Supplementary Table 1), but the confidence intervals were wide and included one. Similarly, liveborn children with healthy grandparents were less likely to die by the age five years.

**Table 3:** Child health outcomes and health service use by the health of their grandparents

	Maternal grandmother			Maternal grandfather				Paternal grandmother				Paternal grandfather			
Child health	Healthy *n* (%)	Unhealthy *n* (%)	Dead *n* (%)	Healthy *n* (%)	Unhealthy *n* (%)	Dead *n* (%)	Unlinked *n* (%)	Healthy *n* (%)	Unhealthy *n* (%)	Dead *n* (%)	Unlinked *n* (%)	Healthy *n* (%)	Unhealthy *n* (%)	Dead *n* (%)	Unlinked *n* (%)
*All births* (*N* = 29,409)															
Stillbirth															
Yes	290 (1)	40 (2)	30 (1)	184 (1)	15 (1)	42 (2)	119 (1)	137 (1)	16 (1)	19 (1)	188 (2)	102 (1)	n.p.	19 (1)	n.p.
No	24,861 (99)	2026 (98)	2162 (99)	16,595 (99)	1064 (99)	2635 (98)	8755 (99)	16,321 (99)	1273 (99)	1521 (99)	9934 (98)	11,601 (99)	n.p.	2045 (99)	n.p.
*Live births* (*N* = 29,049)															
Death before age 5															
Yes	296 (1)	37 (2)	41 (2)	171 (1)	18 (2)	44 (2)	141 (2)	136 (1)	9 (1)	21 (1)	208 (2)	93 (1)	n.p.	20 (1)	n.p.
No	24,565 (99)	1989 (98)	2121 (98)	16,424 (99)	1046 (98)	2591 (98)	8614 (98)	16,185 (99)	1264 (99)	1500 (99)	9726 (98)	11,508 (99)	n.p.	2025 (99)	n.p.
*Alive at 5 years* (*N* = 28,675)															
Total hospital bed days 0–4 years (excluding birth admission)															
Median (IQR)	1 (0–4)	2 (0–6)	2 (0–6)	1 (0–3)	1 (0–4)	1 (0–4)	1 (0–5)	1 (0–3)	1 (0–4)	1 (0–5)	1 (0–5)	1 (0–3)	1 (0–3)	1 (0–4)	1 (0–5)
0	9920 (40)	634 (32)	715 (34)	6991 (43)	417 (40)	1014 (39)	2847 (33)	6622 (41)	461 (36)	509 (34)	3677 (38)	4954 (43)	300 (41)	765 (38)	5250 (36)
1–3	7986 (33)	620 (31)	626 (30)	5496 (33)	337 (32)	827 (32)	2572 (30)	5468 (34)	430 (34)	501 (33)	2833 (29)	3959 (34)	246 (33)	706 (35)	4321 (30)
4–6	2773 (11)	286 (14)	281 (13)	1840 (11)	123 (12)	328 (13)	1049 (12)	1802 (11)	162 (13)	198 (13)	1178 (12)	1218 (11)	82 (11)	239 (12)	1801 (13)
7+	3886 (16)	449 (23)	499 (24)	2097 (13)	169 (16)	422 (16)	2146 (25)	2293 (14)	211 (17)	292 (19)	2038 (21)	1377 (12)	112 (15)	315 (16)	3030 (21)
Total potentially avoidable hospital admissions 0–4 years															
Median (IQR)	0 (0–1)	0 (0–1)	0 (0–1)	0 (0–1)	0 (0–1)	0 (0–1)	0 (0–1)	0 (0–1)	0 (0–1)	0 (0–1)	0 (0–1)	0 (0–1)	0 (0–1)	0 (0–1)	0 (0–1)
0	15,670 (64)	1063 (53)	1214 (57)	11,014 (67)	651 (62)	1636 (63)	4646 (54)	10,558 (65)	786 (62)	864 (58)	5739 (59)	7846 (68)	481 (65)	1302 (64)	8318 (58)
1	5198 (21)	472 (24)	471 (22)	3376 (21)	235 (22)	542 (21)	1988 (23)	3398 (21)	283 (22)	344 (23)	2116 (22)	2310 (20)	157 (21)	428 (21)	3246 (23)
2+	3697 (15)	454 (23)	436 (21)	2034 (12)	160 (15)	413 (16)	1980 (23)	2229 (14)	195 (15)	292 (19)	1871 (19)	1352 (12)	102 (14)	295 (15)	2838 (20)
Any admission for unintentional injury															
No	21,836 (89)	1723 (87)	1866 (88)	14,650 (89)	927 (89)	2304 (89)	7544 (88)	14,365 (89)	1097 (87)	1309 (87)	8654 (89)	10,280 (89)	640 (86)	1773 (88)	12,732 (88)
Yes	2729 (11)	266 (13)	255 (12)	1774 (11)	119 (11)	287 (11)	1070 (12)	1820 (11)	167 (13)	191 (13)	1072 (11)	1228 (11)	100 (14)	252 (12)	1670 (12)
Any unavoidable admissions															
No	23,744 (97)	1909 (96)	2049 (97)	15,901 (97)	1005 (96)	2502 (97)	8294 (96)	15,655 (97)	1218 (96)	1443 (96)	9386 (97)	11,142 (97)	720 (97)	1941 (96)	13,899 (97)
Yes	821 (3)	80 (4)	72 (3)	523 (3)	41 (4)	89 (3)	320 (4)	530 (3)	46 (4)	57 (4)	340 (3)	366 (3)	20 (3)	84 (4)	503 (3)
*Alive at 5 years and born from 2002 onwards* (*N* = 25,021)															
Total ED presentations 0–4 years															
Median (IQR)	5 (2–10)	6 (2–12)	5 (2–11)	5 (2–9)	5 (2–10)	5 (2–9)	5 (2–10)	5 (2–10)	6 (2–11)	5 (2–11)	5 (2–10)	5 (2–9)	5 (2–10)	5 (2–10)	5 (2–11)
0	1691 (8)	120 (7)	152 (8)	1196 (8)	66 (7)	188 (8)	513 (7)	970 (7)	55 (5)	87 (7)	851 (10)	747 (7)	35 (5)	115 (7)	1066 (8)
1–4	7645 (36)	512 (29)	564 (31)	5414 (38)	332 (36)	822 (37)	2153 (29)	5205 (37)	378 (34)	465 (36)	2673 (32)	3889 (39)	238 (36)	657 (38)	3937 (31)
5+	12,092 (56)	1122 (64)	1123 (61)	7766 (54)	533 (57)	1239 (55)	4799 (64)	7963 (56)	684 (61)	749 (58)	4941 (58)	5431 (54)	384 (58)	978 (56)	7544 (60)
Unlinked: The children could not be linked to the grandparent through birth records (Midwives Notification System) or birth registration. *ED*: Emergency Department. *IQR*: Interquartile range. n.p.=counts are not publishable because of privacy concerns as they are less than 5 or could lead to the calculation of a small count

Children with healthy maternal grandmothers spent an average of 27% fewer days in hospital (adjusted incidence rate ratio (aIRR): 0.73, 95% confidence interval (CI): 0.65, 0.83), had 19% fewer potentially avoidable hospital admissions (aIRR: 0.81, 95% CI: 0.74, 0.89) and 8% fewer ED presentations (aIRR: 0.92, 95% CI: 0.87, 0.97), than children with deceased maternal grandmothers. Children with unhealthy maternal grandmothers had similar outcomes to those with deceased grandmothers. Similar patterns were apparent for the other three grandparents. Children with healthy grandparents were less likely to have an admission for unintentional injury. The association was strongest for paternal grandfathers (aIRR: 0.80, 95% CI: 0.69, 0.93). Unavoidable hospital admissions were not associated with the health of any grandparents.

**Fig. 4:**
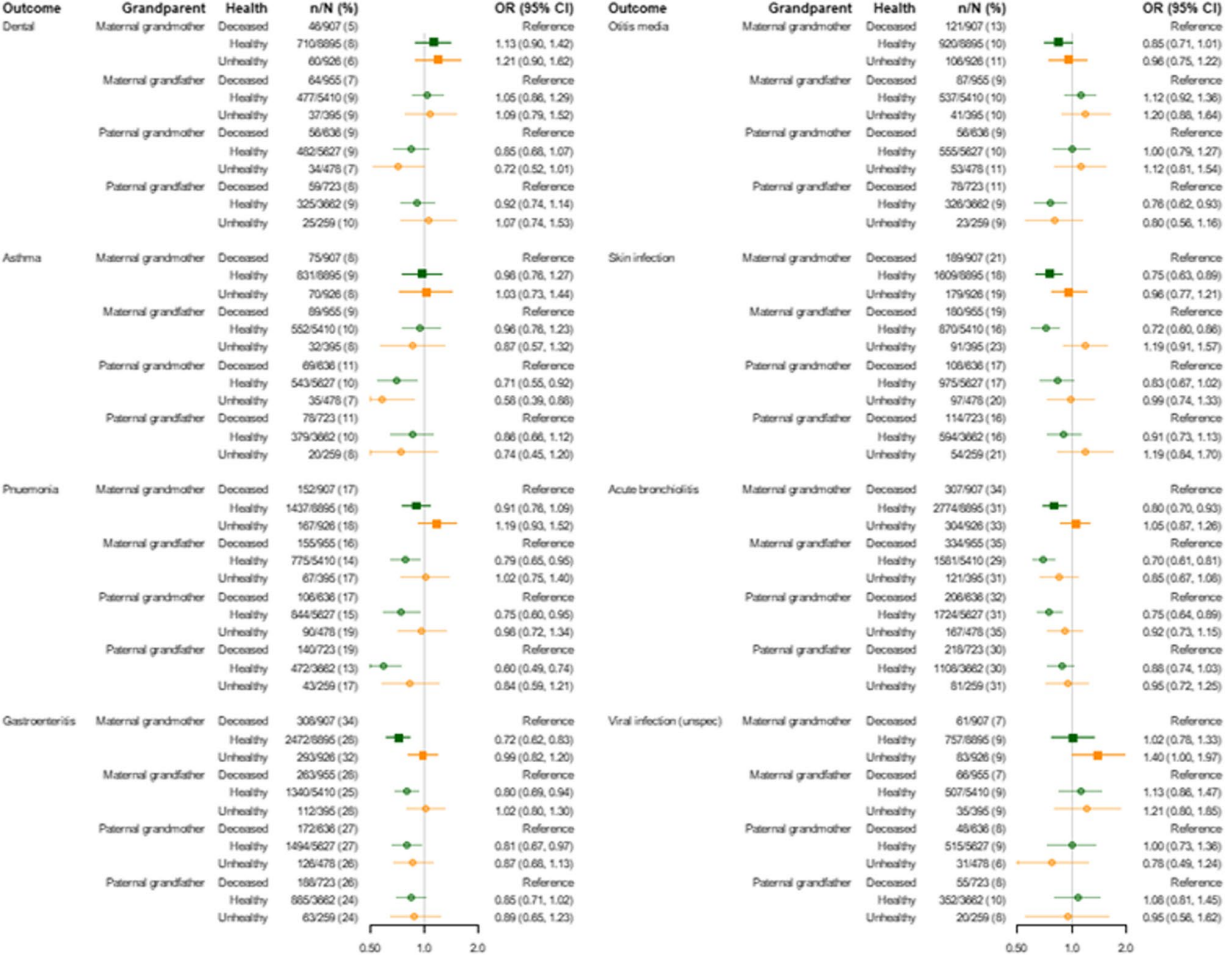
Associations (odds ratios) between children having at least one avoidable hospitalisation by age 5 by category of hospitalisation and grandparental health Models adjusted for the child's year of birth, sex, maternal age, maternal smoking during pregnancy, socioeconomic advantage, and remoteness. OR: Odds ratio. CI: Confidence interval

Adjusting for remoteness, socioeconomic disadvantage, maternal age, and smoking during pregnancy somewhat attenuated the associations with stillbirth, death before age five, total hospital bed days, and potentially avoidable hospital admissions, but slightly increased estimated associations with admissions for unintentional injury and ED presentations (Figs. 3 and 4 and Supplementary Tables 1 and 3). E-values for the fully adjusted estimates for healthy versus deceased maternal grandmothers were 2.08 for hospital bed days and 1.39 for ED presentations (Supplementary Table 2).

Of the main causes of potentially avoidable hospital admission, asthma, pneumonia, gastroenteritis, otitis media, skin infection, and acute bronchiolitis were associated with the health of at least one grandparent (Fig. 4 and Supplementary Table 2). Dental and unspecified viral infection were not.

For the 10, 911 children who linked to all four grandparents, associations were strongest with maternal grandmaternal health for hospital bed days, potentially avoidable hospital admissions, and ED presentations (Supplementary Table 4). However, for admission for unintentional injury, the association was strongest for paternal grandfathers.

The grandparent-child health associations were greater for children with Aboriginal grandmothers than non-Aboriginal grandmothers. This was particularly apparent for potentially avoidable hospital admissions; children with healthy Aboriginal maternal grandmothers had 19% fewer admissions than those with deceased grandmothers (aIRR (95% CI): 0.81 (0.74, 0.89), but there was little evidence of an association for non-Aboriginal grandmothers (aIRR (95% CI): 1.15 (0.85, 1.56)) (Supplementary Table 5).

Associations were also generally stronger among children from major cities and the most advantaged socioeconomic quartile than children from very remote areas and the most disadvantaged quartile, but there was considerable overlap in the confidence intervals (Supplementary Tables 6 and 7). There was no consistent pattern of better outcomes for children who lived in the same region as their maternal grandmothers (Supplementary Table 8).

Child-grandparental health associations were stronger for grandparents with fewer grandchildren (Supplementary Table 9). Children who did not share their maternal grandmother with any siblings or cousins (by age five) had an estimated 38% fewer bed days if their grandmother was healthy compared to those with a deceased grandmother (aIRR 0.62, 95% CI: 0.52, 0.73). However, among those whose maternal grandmothers had ten grandchildren, only 23% fewer bed days were estimated for children with healthy grandmothers, compared to those with deceased grandmothers (aIRR 0.77, 95% CI: 0.68, 0.87).

Results from the first sensitivity analysis with children who had been formally placed in out-of-home care at least once were not meaningfully different from results for the full sample (Supplementary Table 9). After restricting to children whose grandparents had at least one health record in the decade before their birth (the second sensitivity analysis), grandparental-child health associations were slightly stronger than for the main analyses (Supplementary Table 10). For example, the fully adjusted IRR for total hospital bed days was 0.69 (95% CI: 0.61, 0.78) for children with healthy maternal grandmothers compared to those with deceased grandmothers in the sensitivity analysis and 0.73 (95% CI: 0.65, 0.83) in the main analysis. For the other grandparents, point estimates were similar in the main and sensitivity analyses, with no consistent pattern upwards or downwards.

Child health service use was generally higher for grandparents with higher Elixhauser scores (Supplementary Table 11). 41% of children had no hospital admissions in the first five years of life if their maternal grandmother had no comorbidities, compared to 36% of children for one comorbidity, 33% of children for two comorbidities, and 31% for three or more comorbidities.

## Discussion

Aboriginal children with healthy grandparents had lower mortality, fewer hospital bed days, fewer potentially avoidable hospital admissions, and fewer ED presentations in their first five years of life than children with deceased or unhealthy grandparents. These associations were strongest when considering maternal grandmothers and grandparents with fewer grandchildren. By contrast, admissions for unintentional injury were related to the health of paternal grandparents, but not maternal grandparents. Stillbirth and hospital admissions that were not potentially avoidable were unrelated to grandparental health.

The evidence for lower mortality from birth to age five years for children with healthy grandparents was not strong (adjusted OR: 0.71 (0.50, 1.01)). However, it was consistent with the small majority of studies that found positive associations between child survival and grandmaternal survival, studies largely based on pre-industrial populations or more recent populations in the global south [11]. Prior studies on child physical health have had mixed results; care from grandparents was associated with positive impacts on height, weight, and injury in some studies and populations, but negative impacts on body mass index, physical development, and physical function in other studies and populations [10]. We found no evidence that living grandparents were adversely associated with child health in the first five years of life; instead, we found children with living grandparents spent less time in hospital and had fewer ED presentations.

These associations may be causal – care, knowledge, and other supports from healthy grandparents may safeguard the health of young children. Conversely, deceased grandparents cannot care for their grandchildren at all. Unhealthy grandparents may be less able to take care of others than healthy grandparents and may themselves require care. Supporting a causal explanation, associations were observed for outcomes that are more amenable to intervention by carers (for example, potentially avoidable admissions), but were generally absent for outcomes where support was less likely to have an impact, such as stillbirth and unavoidable hospital admissions. The finding that the grandparent-child health association was weaker for children from large extended families may also support a causal explanation; for those grandparents, not only health but also time restraints and other resources may affect how much support they can offer individual children, while for grandparents with few grandchildren, health may be a more important limiting factor. In Aboriginal communities (and many others), maternal grandmothers tend to provide more care for their grandchildren than the other grandparents [4, 36] and if grandparental care fosters good child health, the influence of maternal grandmothers would be greater than for other grandparents. Supporting this hypothesis, stronger child-grandparental health associations were observed for maternal grandmothers than the other grandparents among children who linked to all four grandparents.

However, another explanation for the relationships we observed is that we did not sufficiently control for confounding environmental influences shared by the children and their grandparents. For example, if grandparents and grandchildren both live in an area with inadequate primary health care, grandparents may be more likely to die young or have poor health and the grandchildren may be more likely to be admitted to hospital for conditions that could have been prevented with appropriate primary health care. We controlled for the impact of shared environment by adjusting for area-level socioeconomic deprivation, remoteness, and proxies for individual-level socioeconomic deprivation in maternal smoking during pregnancy and maternal age. This adjustment resulted in limited changes in our estimates, even for outcomes particularly associated with disadvantage like skin infection [37]. The E-value for the IRR of hospital bed days for children with healthy maternal grandmothers compared to those with deceased maternal grandmothers (2.08) indicates that an unmeasured confounder could only explain away the association if it was (1) associated with a doubling of child bed days and (2) twice as common among children with deceased grandparents than children with healthy grandparents. By contrast, the E-value for the IRR for ED presentations (1.39), suggests only a limited amount of unmeasured confounding could explain that association with maternal grandmaternal health. Possible unmeasured confounders could include family functioning and social support networks, cultural influences, grandparental mental health, and effects of intergenerational trauma that impact both grandparental and child health.

Finally, shared genes may explain some of the observed relationships between grandparental and child health. However, this is unlikely to be an important factor, as young children are largely free from the major causes of adult mortality and morbidity.

Complicating a causal explanation for the difference in outcomes for children with healthy and children with unhealthy grandparents (but not the comparison involving children with deceased grandparents) is that healthy grandparents are more likely than unhealthy grandparents to work and they may have less time to care for grandchildren. However, it is likely that many of the healthy grandparents did not work – while over 80% of grandparents were categorised as healthy, a smaller percent of Aboriginal adults was employed. Among those aged 40 to 69 years in WA, fewer than half were recorded as working in the 2021 Census (43% of females and 46% of males were employed and 23% of females and 33% of males were in full-time employment) [38]. Irrespective of whether the associations are causal or not, many implications are the same. Health problems in multiple generations simultaneously may reduce the time, energy, and resources that can be devoted to each generation. Healthy ageing in older Aboriginal people, family-centred approaches, and support for strong intergenerational relationships may improve child health and reduce pressure on families with sick children.

Healthy ageing requires addressing the social determinants of health that underlie the mortality and health gaps between Aboriginal and non-Aboriginal adults. While there has been progress in some areas, (e.g. an increase in employment among people aged 25 to 64 years), others continue to worsen (e.g. incarceration of adults) [39] and more effective commitments from Australian governments and institutions are needed to make progress. Beneficial cultural determinants of health and wellbeing include connections across generations [40]. For older Aboriginal people fulfilling the role of transmitting cultural knowledge to younger generations, caring for family and community, and being respected and valued by younger community members are components of healthy ageing [41, 42]. However, the obligations to family and kin expected of older Aboriginal people may be higher than for non-Aboriginal people and may adversely affect their health [43]. 

More proximate causes of healthy and unhealthy ageing identified by older Aboriginal Australians include access to transport to attend appointments, health literacy, skills using technology (for example, for telehealth), minimising health issues because of concerns about being a burden on the family, housing that is safe in extreme heat and cold, and culturally safe health care [41, 42]. Many older Aboriginal Australians have a preference for Aboriginal Community Controlled Health Organisations (ACCHOs) [44] and the number of ACCHOs has increased from one in 1971 to over 140 today [45]. However, a third (33%) of Aboriginal Western Australians live in remote and very remote areas [46], where the closest health service may not have a full suite of health professionals. For example, in 2020, across all of Australia’s remote and very remote areas, there were only 159 (full-time equivalent) clinical dental practitioners [47]. 

A family-centred approach, with coordinated support for parents caring for both children and older adults, may help families simultaneously face the challenges of caring for children and grandparents with poor health at the same time. Planning for family-centred care is holistic care and support is at the level of the entire family, rather than at the level of individual family members [48]. This approach has been embedded in some primary care programs managed by ACCHOs, with reports of improved child and caregiver health and wellbeing [48]. 

Strong relationships between children and their grandparents are particularly important in Aboriginal communities, where many grandparents are primary carers of their grandchildren, because of the very high rates of removal of Aboriginal children by child protection authorities. By the age of five, 8% of Aboriginal children in WA have been placed in formal out-of-home care at least once [49], with 54% of children in out-of-home care in WA placed with relatives or kin [50]. Additionally, many more children live separately from their parents, outside of the formal child protection system. Carers outside the formal system receive limited support to meet the needs of the children they care for, including their health needs – AUD 1000 per year per child, with a one-off additional payment in January 2024 of AUD 500 for the first child and AUD 250 for subsequent children [51, 52]. 

The results for the sample of children who had never been placed in out-of-home care by age five were similar to the results for the complete sample. This may not be surprising as children placed in out-of-home care are a heterogeneous group; some would have been placed in temporary or permanent full-time care of a grandparent, while others will have lost contact with some, or all, of their grandparents.

Distance can be a barrier to strong intergenerational relationships. Aboriginal adults are less likely to hold drivers licences than non-Aboriginal adults for reasons such as cost and lack of identity documents [53] and private car ownership may be too expensive [54]. In addition, there are barriers to public or community transport, including no or limited services in some regions and concerns about safety [54]. In the 2014-15 National Aboriginal and Torres Strait Islander Social Survey, only 70% of Aboriginal Western Australians reported that they were easily able to get to places when needed and only 62% had access to a motor vehicle when needed [55]. Future housing policies should enable young families to live close to grandparents.

Strengths of this study include that the datasets cover all of WA and family links could be made for the majority of the Aboriginal children born in WA from 2000 to 2013, and 10,911 children (32%) could be linked to all four of their grandparents. The linked datasets provided a range of information about the families that could be used to better understand the results and to undertake appropriate sensitivity analyses.

A major limitation of this study is that the degree and nature of grandparental involvement in the lives of children is not captured in Australian routinely collected datasets. To overcome this, we used a proxy, assuming that in most families, if a grandparent is alive and if they are well enough, they play some role in the child’s life. Another limitation is that the health of the grandparents is imperfectly captured in hospital data. We may have misclassified as healthy some unhealthy grandparents who attended health services outside WA or those who have poor health, but they did not require or did not access treatment in a hospital. Similarly, we may have misclassified as alive some grandparents who died interstate or before the start of the Western Australian electronic death records in 1969. However, the sensitivity analysis which excluded cases where the grandparent had no health record in WA in the ten years before the birth of the child resulted in larger associations than the main analysis for maternal grandmothers and similar associations for the other grandparents. While the Elixhauser score is a good predictor of mortality and some other health outcomes in a range of populations [56–58], including Aboriginal Australians [59, 60], it may not capture well the health conditions that affect caregiving capacity or Aboriginal concepts of health and wellbeing. However, even if grandparental mortality and Elixhauser score is a poor proxy for grandparental health and involvement, the impact would be a bias towards a null finding.

A large proportion of children could not be linked to all four grandparents. As major reasons for not linking were that their fathers or grandfathers were not included on birth registrations, these children likely had less contact with the unlinked grandparents than those who were linked. These children were also more likely to be from more remote and socioeconomically deprived areas and our sensitivity analyses indicated that the grandparent-child health associations may be weaker in such areas. If all Aboriginal children born in WA had been linked to all of their grandparents, it is possible that the observed associations would have been weaker. Finally, there were indications that the maternal grandmaternal-child health associations were weaker for the 24% of children with a non-Indigenous maternal grandmother. Some children had only one Aboriginal grandparent, and others had four—further investigation of this complexity is warranted.

## Conclusions

Aboriginal children with healthy grandparents had fewer days in hospital and emergency presentations than those with unhealthy or deceased grandparents. Support for healthy ageing and strong family relationships may improve child health and reduce the number of families caring for multiple generations at once.

## Supplementary Information

Supplementary Material 1.

## Data Availability

The data analysed in this study are not publicly available and the authors do not have permission from the data custodians to share the data. However, with relevant ethics permissions and approval of the data custodians of each of the datasets used in this study the data can be obtained from the WA Government. For information about data access, approval from the data custodians and data linkage, researchers can contact the Western Australian Data Linkage Services (https://www.datalinkageservices.health.wa.gov.au/).

## Abbreviations

ACCHO: Aboriginal Community Controlled Health Organisation
aIRR: Adjusted incidence rate ratio
ARIA+: Accessibility/Remoteness Index for Australia
CI: Confidence interval
ED: Emergency Department
ICD-10-AM: International Statistical Classification of Diseases and Related Health Problems, Tenth Revision, Australian Modification
IRR: Incidence rate ratio
IRSEO: Indigenous Relative Socioeconomic Outcomes Index
OR: Odds ratio
SA1: Statistical Area 1
WA: Western Australia
