# Supplementary material for: Health of Aboriginal and Torres Strait Islander children and their grandparents: a Western Australian retrospective cohort study
Source: BMC Public Health. 2025 Oct 9;25:3450. doi: 10.1186/s12889-025-24577-0 (PMC12512576; doi:10.1186/s12889-025-24577-0)
Supplement: Supplementary file 1 — Supplementary Material 1. [file 12889_2025_24577_MOESM1_ESM.docx]

**Supplementary material**

**Supplementary Table 1: Associations between child health outcomes and health service use and grandparental health**

|  |  | **Maternal grandmother** | | |  |  | **Maternal grandfather** | | |  |  | **Paternal grandmother** | | |  |  | **Paternal grandfather** | | |  |  |
| --- | --- | --- | --- | --- | --- | --- | --- | --- | --- | --- | --- | --- | --- | --- | --- | --- | --- | --- | --- | --- | --- |
| **Child health** | **Adjustment** | **Healthy** | **Unhealthy** | **Deceased** | **p** |  | **Healthy** | **Unhealthy** | **Deceased** | **p** |  | **Healthy** | **Unhealthy** | **Deceased** | **p** |  | **Healthy** | **Unhealthy** | **Deceased** | **p** |  |
| *All births with no missing data* | |  | N=29,376 |  |  |  |  | N=20,516 |  |  |  |  | N=19,275 |  |  |  |  | N=14,519 |  |  |  |
| Stillbirth (OR) |  |  |  |  |  |  |  |  |  |  |  |  |  |  |  |  |  |  |  |  |  |
|  | YOB and sex | 0.81 (0.55, 1.18) | 1.35 (0.83, 2.21) | Ref | 0.04 |  | 0.68 (0.48, 0.97) | 0.89 (0.49, 1.62) | Ref | 0.13 |  | 0.68 (0.41, 1.12) | 1.00 (0.50, 1.99) | Ref | 0.21 |  | 0.93 (0.57, 1.52) | 1.28 (0.58, 2.83) | Ref | 0.72 |  |
|  | Fully adjusted | 0.87 (0.59, 1.27) | 1.38 (0.85, 2.26) | Ref | 0.08 |  | 0.71 (0.50, 1.02) | 0.90 (0.49, 1.65) | Ref | 0.20 |  | 0.75 (0.46, 1.25) | 1.09 (0.54, 2.18) | Ref | 0.33 |  | 1.03 (0.63, 1.69) | 1.36 (0.61, 3.03) | Ref | 0.76 |  |
|  |  |  |  |  |  |  |  |  |  |  |  |  |  |  |  |  |  |  |  |  |  |
| *Live births with no missing data* | |  | N=29,019 |  |  |  |  | N=20,277 |  |  |  |  | N=19,103 |  |  |  |  | N=14,389 |  |  |  |
| Death before age 5 (OR) | |  |  |  |  |  |  |  |  |  |  |  |  |  |  |  |  |  |  |  |  |
|  | YOB and sex | 0.63 (0.45, 0.89) | 0.98 (0.61, 1.58) | Ref | 0.02 |  | 0.63 (0.44, 0.89) | 1.01 (0.55, 1.85) | Ref | 0.04 |  | 0.59 (0.37, 0.94) | 0.49 (0.22, 1.09) | Ref | 0.15 |  | 0.80 (0.49, 1.30) | 0.50 (0.13, 1.87) | Ref | 0.44 |  |
|  | Fully adjusted | 0.71 (0.50, 1.01) | 1.04 (0.65, 1.68) | Ref | 0.06 |  | 0.70 (0.48, 1.02) | 1.05 (0.57, 1.94) | Ref | 0.12 |  | 0.66 (0.40, 1.07) | 0.51 (0.23, 1.17) | Ref | 0.23 |  | 0.93 (0.57, 1.51) | 0.55 (0.15, 2.07) | Ref | 0.56 |  |
|  |  |  |  |  |  |  |  |  |  |  |  |  |  |  |  |  |  |  |  |  |  |
| *Alive at 5 years with no missing data* | | | N=28,645 |  |  |  |  | N=20,044 |  |  |  |  | N=18,937 |  |  |  |  | N=14,272 |  |  |  |
| Total hospital bed days 0-4 years (IRR) | | |  |  |  |  |  |  |  |  |  |  |  |  |  |  |  |  |  |  |  |
|  | YOB and sex | 0.67 (0.60, 0.75) | 0.96 (0.81, 1.13) | Ref | <0.001 |  | 0.81 (0.70, 0.93) | 1.03 (0.81, 1.32) | Ref | 0.005 |  | 0.71 (0.59, 0.84) | 0.82 (0.65, 1.04) | Ref | 0.001 |  | 0.73 (0.61, 0.88) | 0.81 (0.62, 1.06) | Ref | 0.011 |  |
|  | Fully adjusted | 0.73 (0.65, 0.83) | 0.95 (0.81, 1.12) | Ref | <0.001 |  | 0.84 (0.73, 0.95) | 0.96 (0.77, 1.18) | Ref | 0.02 |  | 0.68 (0.57, 0.81) | 0.79 (0.63, 0.98) | Ref | <0.001 |  | 0.72 (0.60, 0.88) | 0.77 (0.61, 0.97) | Ref | 0.012 |  |
|  |  |  |  |  |  |  |  |  |  |  |  |  |  |  |  |  |  |  |  |  |  |
| Potentially avoidable hospital admissions 0-4 years (IRR) | | | |  |  |  |  |  |  |  |  |  |  |  |  |  |  |  |  |  |  |
|  | YOB and sex | 0.78 (0.71, 0.85) | 1.00 (0.90, 1.12) | Ref | <0.001 |  | 0.83 (0.77, 0.91) | 1.01 (0.87, 1.17) | Ref | <0.001 |  | 0.77 (0.69, 0.85) | 0.88 (0.76, 1.02) | Ref | <0.001 |  | 0.81 (0.72, 0.91) | 0.88 (0.73, 1.05) | Ref | 0.005 |  |
|  | Fully adjusted | 0.81 (0.74, 0.89) | 1.00 (0.89, 1.12) | Ref | <0.001 |  | 0.82 (0.75, 0.90) | 0.95 (0.82, 1.09) | Ref | <0.001 |  | 0.76 (0.68, 0.85) | 0.86 (0.74, 1.00) | Ref | <0.001 |  | 0.80 (0.71, 0.91) | 0.86 (0.71, 1.04) | Ref | 0.006 |  |
|  |  |  |  |  |  |  |  |  |  |  |  |  |  |  |  |  |  |  |  |  |  |
| Any admission for unintentional injury (OR) | | |  |  |  |  |  |  |  |  |  |  |  |  |  |  |  |  |  |  |  |
|  | YOB and sex | 0.92 (0.80, 1.06) | 1.13 (0.93, 1.36) | Ref | 0.01 |  | 0.98 (0.86, 1.12) | 1.04 (0.83, 1.30) | Ref | 0.81 |  | 0.87 (0.74, 1.02) | 1.05 (0.83, 1.31) | Ref | 0.04 |  | 0.84 (0.73, 0.98) | 1.09 (0.84, 1.40) | Ref | 0.02 |  |
|  | Fully adjusted | 0.89 (0.77, 1.02) | 1.08 (0.89, 1.30) | Ref | 0.01 |  | 0.94 (0.82, 1.08) | 1.01 (0.81, 1.26) | Ref | 0.60 |  | 0.83 (0.70, 0.97) | 0.99 (0.78, 1.24) | Ref | 0.02 |  | 0.80 (0.69, 0.93) | 1.02 (0.79, 1.31) | Ref | 0.006 |  |
| Any unavoidable admissions (OR) | | |  |  |  |  |  |  |  |  |  |  |  |  |  |  |  |  |  |  |  |
|  | YOB and sex | 1.00 (0.78, 1.28) | 1.20 (0.87, 1.67) | Ref | 0.34 |  | 0.94 (0.74, 1.20) | 1.18 (0.79, 1.75) | Ref | 0.47 |  | 0.87 (0.66, 1.16) | 0.96 (0.64, 1.43) | Ref | 0.58 |  | 0.77 (0.60, 0.99) | 0.66 (0.40, 1.09) | Ref | 0.12 |  |
|  | Fully adjusted | 1.00 (0.78, 1.29) | 1.20 (0.86, 1.66) | Ref | 0.39 |  | 0.92 (0.72, 1.17) | 1.15 (0.77, 1.72) | Ref | 0.40 |  | 0.88 (0.66, 1.17) | 0.96 (0.64, 1.43) | Ref | 0.62 |  | 0.78 (0.60, 1.00) | 0.67 (0.41, 1.11) | Ref | 0.14 |  |
|  |  |  |  |  |  |  |  |  |  |  |  |  |  |  |  |  |  |  |  |  |  |
| *Alive at 5 years, born from 2002 onwards and no missing data* | | | N=24,998 |  |  |  |  | N=17,545 |  |  |  |  | N=16,545 |  |  |  |  | N=12,473 |  |  |  |
| ED presentations 0-4 years (IRR) | |  |  |  |  |  |  |  |  |  |  |  |  |  |  |  |  |  |  |  |  |
|  | YOB and sex | 0.92 (0.87, 0.97) | 1.04 (0.97, 1.11) | Ref | <0.001 |  | 0.99 (0.93, 1.04) | 1.01 (0.93, 1.09) | Ref | 0.69 |  | 0.93 (0.86, 0.99) | 1.02 (0.94, 1.11) | Ref | <0.001 |  | 0.94 (0.87, 1.01) | 0.96 (0.88, 1.05) | Ref | 0.28 |  |
|  | Fully adjusted | 0.92 (0.87, 0.97) | 1.02 (0.96, 1.09) | Ref | <0.001 |  | 0.93 (0.88, 0.98) | 0.93 (0.86, 1.01) | Ref | 0.04 |  | 0.90 (0.84, 0.96) | 0.99 (0.91, 1.07) | Ref | <0.001 |  | 0.91 (0.84, 0.98) | 0.94 (0.85, 1.04) | Ref | 0.05 |  |
| p: P-value. YOB: Year of birth. OR: odds ratio with 95% confidence interval. IRR: incidence rate ratio with 95% confidence interval. YOB: year of birth. Fully adjusted estimates were obtained from models that included child sex, year of birth, and components of the child's environment: remoteness, socioeconomic disadvantage, maternal age, and maternal smoking during pregnancy. 33 children were excluded because of incomplete covariate data leaving 29,376 children contributing to the first model. Each estimate is from a separate model, with the health of only one grandparent included in each model. | | | | | | | | | | | | | | | | | | | | | |

**Supplementary Table 2: E-values for the adjusted associations between child health outcomes and health service use and grandparental health**

| Child health | Grandparent | Estimate (95% CI) | E-value for estimate | E-value for 95% CI |
| --- | --- | --- | --- | --- |
| Total hospital bed days 0-4 years (excluding birth admission) | Maternal grandmother | 0.73 (0.65, 0.83) | 2.08 | 1.70 |
|  | Maternal grandfather | 0.84 (0.73, 0.95) | 1.67 | 1.29 |
|  | Paternal grandmother | 0.68 (0.57, 0.81) | 2.30 | 1.77 |
|  | Paternal grandfather | 0.72 (0.60, 0.88) | 2.12 | 1.53 |
| Total potentially avoidable hospital admissions 0-4 years | Maternal grandmother | 0.81 (0.74, 0.89) | 1.77 | 1.50 |
|  | Maternal grandfather | 0.82 (0.75, 0.90) | 1.74 | 1.46 |
|  | Paternal grandmother | 0.76 (0.68, 0.85) | 1.96 | 1.63 |
|  | Paternal grandfather | 0.80 (0.71, 0.91) | 1.81 | 1.43 |
| Any admission for unintentional injury | Paternal grandmother | 0.83 (0.70, 0.97) | 1.70 | 1.21 |
|  | Paternal grandfather | 0.80 (0.69, 0.93) | 1.81 | 1.36 |
| Total ED presentations 0-4 years | Maternal grandmother | 0.92 (0.87, 0.97) | 1.39 | 1.21 |
|  | Maternal grandfather | 0.93 (0.88, 0.98) | 1.36 | 1.16 |
|  | Paternal grandmother | 0.90 (0.84, 0.96) | 1.46 | 1.25 |
|  | Paternal grandfather | 0.91 (0.84, 0.98) | 1.43 | 1.16 |
| CI: Confidence interval. ED: Emergency department. Associations with CIs that included 1 were not included. | | | | |

**Supplementary Table 3: Associations (odds ratios) between children having at least one avoidable hospitalisation by age 5 by category of hospitalisation and grandparental health**

|  |  | **Maternal grandmother (N=28,645)** | |  | |  | **Maternal grandfather (N=20,044)** | |  | |  | **Paternal grandmother (N=18,937)** | |  | |  | **Paternal grandfather (N=14,272)** | |  | |
| --- | --- | --- | --- | --- | --- | --- | --- | --- | --- | --- | --- | --- | --- | --- | --- | --- | --- | --- | --- | --- |
| **Child health** | **Adjustment** | **Healthy** | **Unhealthy** | **Deceased** | **p** |  | **Healthy** | **Unhealthy** | **Deceased** | **p** |  | **Healthy** | **Unhealthy** | **Deceased** | **p** |  | **Healthy** | **Unhealthy** | **Deceased** | **p** |
| Dental |  |  |  |  |  |  |  |  |  |  |  |  |  |  |  |  |  |  |  |  |
|  | YOB and sex | 1.16 (0.92, 1.45) | 1.21 (0.90, 1.62) | Ref | 0.36 |  | 1.06 (0.86, 1.29) | 1.09 (0.78, 1.51) | Ref | 0.84 |  | 0.87 (0.69, 1.09) | 0.74 (0.53, 1.03) | Ref | 0.19 |  | 0.93 (0.75, 1.16) | 1.07 (0.75, 1.53) | Ref | 0.60 |
|  | Fully adjusted | 1.13 (0.90, 1.42) | 1.21 (0.90, 1.62) | Ref | 0.42 |  | 1.05 (0.86, 1.29) | 1.09 (0.79, 1.52) | Ref | 0.84 |  | 0.85 (0.68, 1.07) | 0.72 (0.52, 1.01) | Ref | 0.16 |  | 0.92 (0.74, 1.14) | 1.07 (0.74, 1.53) | Ref | 0.53 |
| Asthma |  |  |  |  |  |  |  |  |  |  |  |  |  |  |  |  |  |  |  |  |
|  | YOB and sex | 0.97 (0.75, 1.24) | 1.03 (0.74, 1.44) | Ref | 0.87 |  | 0.96 (0.76, 1.22) | 0.88 (0.58, 1.34) | Ref | 0.84 |  | 0.70 (0.54, 0.91) | 0.59 (0.39, 0.89) | Ref | 0.03 |  | 0.85 (0.65, 1.10) | 0.73 (0.45, 1.18) | Ref | 0.34 |
|  | Fully adjusted | 0.98 (0.76, 1.27) | 1.03 (0.73, 1.44) | Ref | 0.93 |  | 0.96 (0.76, 1.23) | 0.87 (0.57, 1.32) | Ref | 0.80 |  | 0.71 (0.55, 0.92) | 0.58 (0.39, 0.88) | Ref | 0.03 |  | 0.86 (0.66, 1.12) | 0.74 (0.45, 1.20) | Ref | 0.39 |
| Bacterial/unspecified pneumonia | | |  |  |  |  |  |  |  |  |  |  |  |  |  |  |  |  |  |  |
|  | YOB and sex | 0.82 (0.68, 0.98) | 1.17 (0.92, 1.49) | Ref | <0.001 |  | 0.80 (0.66, 0.97) | 1.11 (0.81, 1.51) | Ref | 0.02 |  | 0.73 (0.58, 0.91) | 0.99 (0.73, 1.35) | Ref | 0.003 |  | 0.58 (0.48, 0.71) | 0.87 (0.61, 1.24) | Ref | <0.001 |
|  | Fully adjusted | 0.91 (0.76, 1.09) | 1.19 (0.93, 1.52) | Ref | 0.02 |  | 0.79 (0.65, 0.95) | 1.02 (0.75, 1.40) | Ref | 0.02 |  | 0.75 (0.60, 0.95) | 0.98 (0.72, 1.34) | Ref | 0.01 |  | 0.60 (0.49, 0.74) | 0.84 (0.59, 1.21) | Ref | <0.001 |
| Gastroenteritis | |  |  |  |  |  |  |  |  |  |  |  |  |  |  |  |  |  |  |  |
|  | YOB and sex | 0.67 (0.58, 0.77) | 0.98 (0.82, 1.19) | Ref | <0.001 |  | 0.82 (0.70, 0.95) | 1.08 (0.85, 1.38) | Ref | 0.005 |  | 0.79 (0.66, 0.94) | 0.89 (0.69, 1.14) | Ref | 0.03 |  | 0.84 (0.71, 1.00) | 0.92 (0.68, 1.26) | Ref | 0.16 |
|  | Fully adjusted | 0.72 (0.62, 0.83) | 0.99 (0.82, 1.20) | Ref | <0.001 |  | 0.80 (0.69, 0.94) | 1.02 (0.80, 1.30) | Ref | 0.006 |  | 0.81 (0.67, 0.97) | 0.87 (0.68, 1.13) | Ref | 0.08 |  | 0.85 (0.71, 1.02) | 0.89 (0.65, 1.23) | Ref | 0.23 |
| Otitis media |  |  |  |  |  |  |  |  |  |  |  |  |  |  |  |  |  |  |  |  |
|  | YOB and sex | 0.80 (0.67, 0.95) | 0.93 (0.73, 1.18) | Ref | 0.02 |  | 1.09 (0.90, 1.32) | 1.18 (0.86, 1.61) | Ref | 0.54 |  | 0.98 (0.78, 1.24) | 1.10 (0.80, 1.51) | Ref | 0.67 |  | 0.77 (0.63, 0.94) | 0.80 (0.55, 1.16) | Ref | 0.06 |
|  | Fully adjusted | 0.85 (0.71, 1.01) | 0.96 (0.75, 1.22) | Ref | 0.13 |  | 1.12 (0.92, 1.36) | 1.20 (0.88, 1.64) | Ref | 0.42 |  | 1.00 (0.79, 1.27) | 1.12 (0.81, 1.54) | Ref | 0.69 |  | 0.76 (0.62, 0.93) | 0.80 (0.56, 1.16) | Ref | 0.05 |
| Skin infection |  |  |  |  |  |  |  |  |  |  |  |  |  |  |  |  |  |  |  |  |
|  | YOB and sex | 0.71 (0.60, 0.84) | 0.98 (0.79, 1.23) | Ref | <0.001 |  | 0.75 (0.63, 0.90) | 1.29 (0.99, 1.69) | Ref | <0.001 |  | 0.83 (0.68, 1.03) | 1.03 (0.77, 1.38) | Ref | 0.07 |  | 0.93 (0.75, 1.16) | 1.26 (0.89, 1.79) | Ref | 0.19 |
|  | Fully adjusted | 0.75 (0.63, 0.89) | 0.96 (0.77, 1.21) | Ref | <0.001 |  | 0.72 (0.60, 0.86) | 1.19 (0.91, 1.57) | Ref | <0.001 |  | 0.83 (0.67, 1.02) | 0.99 (0.74, 1.33) | Ref | 0.10 |  | 0.91 (0.73, 1.13) | 1.19 (0.84, 1.70) | Ref | 0.21 |
| Acute bronchiolitis | |  |  |  |  |  |  |  |  |  |  |  |  |  |  |  |  |  |  |  |
|  | YOB and sex | 0.78 (0.68, 0.90) | 1.07 (0.89, 1.28) | Ref | <0.001 |  | 0.73 (0.64, 0.84) | 0.91 (0.72, 1.15) | Ref | <0.001 |  | 0.76 (0.64, 0.89) | 0.95 (0.76, 1.19) | Ref | <0.001 |  | 0.89 (0.75, 1.04) | 1.00 (0.76, 1.32) | Ref | 0.26 |
|  | Fully adjusted | 0.80 (0.70, 0.93) | 1.05 (0.87, 1.26) | Ref | <0.001 |  | 0.70 (0.61, 0.81) | 0.85 (0.67, 1.08) | Ref | <0.001 |  | 0.75 (0.64, 0.89) | 0.92 (0.73, 1.15) | Ref | 0.002 |  | 0.88 (0.74, 1.03) | 0.95 (0.72, 1.25) | Ref | 0.28 |
| Viral infection of unspecified site | | |  |  |  |  |  |  |  |  |  |  |  |  |  |  |  |  |  |  |
|  | YOB and sex | 1.05 (0.80, 1.37) | 1.44 (1.03, 2.01) | Ref | 0.05 |  | 1.18 (0.91, 1.54) | 1.24 (0.81, 1.88) | Ref | 0.40 |  | 1.01 (0.74, 1.37) | 0.80 (0.50, 1.27) | Ref | 0.38 |  | 1.13 (0.85, 1.51) | 0.98 (0.58, 1.66) | Ref | 0.60 |
|  | Fully adjusted | 1.02 (0.78, 1.33) | 1.40 (1.00, 1.97) | Ref | 0.05 |  | 1.13 (0.86, 1.47) | 1.21 (0.80, 1.85) | Ref | 0.59 |  | 1.00 (0.73, 1.36) | 0.78 (0.49, 1.24) | Ref | 0.36 |  | 1.08 (0.81, 1.45) | 0.95 (0.56, 1.62) | Ref | 0.75 |
| p: P-value. YOB: Year of birth Fully adjusted estimates were obtained from models which included child sex, year of birth, and components of the child's environment: remoteness, socioeconomic disadvantage, maternal age, and maternal smoking during pregnancy. 30 children were excluded because of incomplete covariate data leaving 28,645 children contributing to the first model. Each estimate is from a separate model, with the health of only one grandparent included in each model. | | | | | | | | | | | | | | | | | | | | |

**Supplementary Table 4: Associations between child health outcomes and health service use and grandparental health among children linked to all 4 grandparents**

| **Child health** | **Grandparental health** | **Maternal grandmother** | **p** | **Maternal grandfather** | **p** | **Paternal grandmother** | **p** | **Paternal grandfather** | **p** |
| --- | --- | --- | --- | --- | --- | --- | --- | --- | --- |
| *Alive at 5 years with no missing data (N=10,911)* | | |  |  |  |  |  |  |  |
| Total hospital bed days (IRR) | |  | 0.04 |  | 0.35 |  | 0.03 |  | 0.07 |
|  | Deceased | Ref |  | Ref |  | Ref |  | Ref |  |
|  | Healthy | 0.67 (0.52, 0.87) |  | 0.87 (0.72, 1.05) |  | 0.73 (0.58, 0.93) |  | 0.75 (0.60, 0.94) |  |
|  | Unhealthy | 0.69 (0.51, 0.92) |  | 0.94 (0.70, 1.27) |  | 0.93 (0.65, 1.32) |  | 0.82 (0.62, 1.07) |  |
|  |  |  |  |  |  |  |  |  |  |
| Potentially avoidable hospital admissions 0-4 years (IRR) | | | 0.01 |  | 0.02 |  | 0.03 |  | 0.04 |
|  | Deceased | Ref |  | Ref |  | Ref |  | Ref |  |
|  | Healthy | 0.71 (0.58, 0.88) |  | 0.83 (0.72, 0.94) |  | 0.78 (0.65, 0.94) |  | 0.80 (0.68, 0.94) |  |
|  | Unhealthy | 0.81 (0.62, 1.05) |  | 0.93 (0.75, 1.14) |  | 0.90 (0.71, 1.16) |  | 0.85 (0.68, 1.07) |  |
|  |  |  |  |  |  |  |  |  |  |
| Any admission for unintentional injury (OR) | | | 0.25 |  | 0.17 |  | 0.26 |  | 0.005 |
|  | Deceased | Ref |  | Ref |  | Ref |  | Ref |  |
|  | Healthy | 0.88 (0.67, 1.15) |  | 1.05 (0.85, 1.29) |  | 0.89 (0.70, 1.13) |  | 0.82 (0.68, 0.98) |  |
|  | Unhealthy | 1.08 (0.75, 1.55) |  | 1.36 (0.99, 1.87) |  | 1.07 (0.78, 1.47) |  | 1.22 (0.90, 1.64) |  |
|  |  |  |  |  |  |  |  |  |  |
| *Alive at 5 years, born from 2002 onwards and no missing data (N=9,554)* | | | | |  |  |  |  |  |
| ED presentations 0-4 years (IRR) | |  | <0.001 |  | 0.02 |  | 0.02 |  | 0.06 |
|  | Deceased | Ref |  | Ref |  | Ref |  | Ref |  |
|  | Healthy | 0.84 (0.76, 0.93) |  | 0.90 (0.83, 0.97) |  | 0.95 (0.86, 1.04) |  | 0.89 (0.80, 0.98) |  |
|  | Unhealthy | 0.94 (0.83, 1.06) |  | 0.91 (0.82, 1.02) |  | 1.06 (0.95, 1.19) |  | 0.94 (0.83, 1.06) |  |
| p: P-value. OR: odds ratio with 95% confidence interval. IRR: incidence rate ratio with 95% confidence interval. Adjusted estimates were obtained from models that included child sex, year of birth, and components of the child's environment: remoteness, socioeconomic disadvantage, maternal age, and maternal smoking during pregnancy. One child was excluded because of incomplete covariate data leaving 10,911 children contributing to the first model. | | | | | | | | | |

**Supplementary Table 5: Associations between child health outcomes and health service use and maternal grandmaternal health by Aboriginal status of their maternal grandmother**

|  |  |  | | |  |
| --- | --- | --- | --- | --- | --- |
| **Child health** | **Aboriginal status of maternal grandmother** | **Healthy** | **Unhealthy** | **Deceased** | **P** |
| *Alive at 5 years with no missing data (N=27,701)* | |  |  |  |  |
| Total hospital bed days (IRR)* |  |  |  |  | 0.37 |
|  | Aboriginal | 0.72 (0.64, 0.82) | 0.92 (0.77, 1.08) | Ref |  |
|  | Non-Aboriginal | 0.94 (0.65, 1.36) | 1.17 (0.63, 2.16) | Ref |  |
|  |  |  |  |  |  |
| Potentially avoidable hospital admissions 0-4 years (IRR) | | |  |  | 0.05 |
|  | Aboriginal | 0.81 (0.74, 0.89) | 0.96 (0.86, 1.08) | Ref |  |
|  | Non-Aboriginal | 1.15 (0.85, 1.56) | 1.39 (0.90, 2.16) | Ref |  |
|  |  |  |  |  |  |
| Any admission for unintentional injury (OR) | |  |  |  | 0.34 |
|  | Aboriginal | 0.90 (0.77, 1.04) | 1.02 (0.84, 1.24) | Ref |  |
|  | Non-Aboriginal | 1.13 (0.70, 1.85) | 1.77 (0.89, 3.52) | Ref |  |
|  |  |  |  |  |  |
| *Alive at 5 years, born from 2002 onwards and no missing data (N=24,357)* | | |  |  |  |
| ED presentations 0-4 years (IRR) |  |  |  |  | 0.32 |
|  | Aboriginal | 0.93 (0.88, 0.99) | 1.02 (0.95, 1.09) | Ref |  |
|  | Non-Aboriginal | 1.05 (0.89, 1.23) | 1.09 (0.89, 1.33) | Ref |  |
| P-values are for interactions between maternal grandmaternal health and Aboriginal status. OR: odds ratio with 95% confidence interval. IRR: incidence rate ratio with 95% confidence interval. Adjusted estimates were obtained from models that included child sex, year of birth, and components of the child's environment: remoteness, socioeconomic disadvantage, maternal age, and maternal smoking during pregnancy. 1012 children were excluded because of incomplete covariate data leaving 28,397 children contributing to the first model. | | | | | |

**Supplementary Table 6: Associations between child health outcomes and health service use and maternal grandmaternal health by maternal socioeconomic status**

| **Child health** | **Maternal socioeconomic disadvantage (quartiles)** | **Healthy** | **Unhealthy** | **Deceased** | **p** |
| --- | --- | --- | --- | --- | --- |
| *Alive at 5 years with no missing data (N=28,645)* | |  |  |  |  |
| Total hospital bed days (aIRR)* |  |  |  |  | 0.60 |
|  | Most advantaged | 0.69 (0.49, 0.98) | 1.02 (0.67, 1.57) | Ref |  |
|  | Least advantaged | 0.80 (0.68, 0.94) | 1.04 (0.83, 1.30) | Ref |  |
|  |  |  |  |  |  |
| Potentially avoidable hospital admissions 0-4 years (aIRR) | |  |  |  | 0.78 |
|  | Most advantaged | 0.79 (0.65, 0.97) | 1.01 (0.77, 1.33) | Ref |  |
|  | Least advantaged | 0.88 (0.76, 1.02) | 1.06 (0.89, 1.27) | Ref |  |
|  |  |  |  |  |  |
| Any admission for unintentional injury (aOR) | |  |  |  | 0.31 |
|  | Most advantaged | 0.66 (0.50, 0.87) | 0.74 (0.50, 1.10) | Ref |  |
|  | Least advantaged | 0.93 (0.72, 1.19) | 1.04 (0.74, 1.44) | Ref |  |
|  |  |  |  |  |  |
| *Alive at 5 years, born from 2002 onwards and no missing data (N=24,998)* | | |  |  |  |
| ED presentations 0-4 years (aIRR) |  |  |  |  | 0.38 |
|  | Most advantaged | 0.88 (0.79, 0.98) | 0.94 (0.82, 1.08) | Ref |  |
|  | Least advantaged | 0.97 (0.89, 1.06) | 1.06 (0.96, 1.19) | Ref |  |
| The P-value is for the interaction between maternal grandmaternal health and maternal socioeconomic disadvantage at the time of the child's birth. aOR: adjusted odds ratio with 95% confidence interval. aIRR: adjusted incidence rate ratio with 95% confidence interval. Adjusted estimates were obtained from models that included child sex, year of birth, and components of the child's environment: remoteness, socioeconomic disadvantage, maternal age, and maternal smoking during pregnancy. 30 children were excluded because of incomplete covariate data leaving 28,645 children contributing to the first model. | | | | | |

**Supplementary Table 7: Associations between child health outcomes and health service use and maternal grandmaternal health by remoteness of residence at birth**

| **Child health** | **Remoteness of child's place of residence at birth** | **Healthy** | **Unhealthy** | **Deceased** | **P** |
| --- | --- | --- | --- | --- | --- |
| *Alive at 5 years with no missing data (N=28,645)* | |  |  |  |  |
| Total hospital bed days (aIRR)* |  |  |  |  | 0.26 |
|  | Major cities | 0.76 (0.59, 0.97) | 0.96 (0.71, 1.30) | Ref |  |
|  | Very remote | 0.71 (0.58, 0.88) | 0.99 (0.73, 1.33) | Ref |  |
|  |  |  |  |  |  |
| Potentially avoidable hospital admissions 0-4 years (aIRR) | |  |  |  | 0.89 |
|  | Major cities | 0.77 (0.65, 0.90) | 0.96 (0.78, 1.18) | Ref |  |
|  | Very remote | 0.86 (0.75, 0.99) | 1.02 (0.85, 1.23) | Ref |  |
|  |  |  |  |  |  |
| Any admission for unintentional injury (aOR) | |  |  |  | 0.24 |
|  | Major cities | 0.76 (0.61, 0.94) | 0.91 (0.67, 1.22) | Ref |  |
|  | Very remote | 0.83 (0.61, 1.11) | 0.93 (0.62, 1.38) | Ref |  |
|  |  |  |  |  |  |
| *Alive at 5 years, born from 2002 onwards and no missing data (N=24,998)* | |  |  |  |  |
| ED presentations 0-4 years (aIRR) |  |  |  |  | 0.73 |
|  | Major cities | 0.90 (0.82, 0.98) | 0.97 (0.87, 1.09) | Ref |  |
|  | Very remote | 0.97 (0.87, 1.08) | 1.06 (0.93, 1.21) | Ref |  |
| P for interaction between maternal grandmaternal health and number of grandchildren. aOR: adjusted odds ratio with 95% confidence interval. aIRR: adjusted incidence rate ratio with 95% confidence interval. Adjusted estimates were obtained from models that included child sex, year of birth, and components of the child's environment: remoteness, socioeconomic disadvantage, maternal age, and maternal smoking during pregnancy. 30 children were excluded because of incomplete covariate data leaving 28,645 children contributing to the first model. | | | | | |

**Supplementary Table 8: Associations between child health outcomes and health service use and maternal grandmaternal health by whether they lived in the same region**

| **Child health** | **Residence of child and grandparent** | **Healthy** | **Unhealthy** | **p** |
| --- | --- | --- | --- | --- |
| *Alive at 5 years with no missing data (N=21,823)* | |  |  |  |
| Total hospital bed days (aIRR)* |  |  |  | 0.06 |
|  | Same region | 0.71 (0.61, 0.82) | Ref |  |
|  | Different region | 0.90 (0.74, 1.11) | Ref |  |
|  |  |  |  |  |
| Potentially avoidable hospital admissions 0-4 years (aIRR) | |  |  | 0.96 |
|  | Same region | 0.81 (0.74, 0.89) | Ref |  |
|  | Different region | 0.81 (0.69, 0.95) | Ref |  |
|  |  |  |  |  |
| Any admission for unintentional injury (aOR) | |  |  | 0.72 |
|  | Same region | 0.84 (0.71, 0.99) | Ref |  |
|  | Different region | 0.79 (0.60, 1.04) | Ref |  |
|  |  |  |  |  |
| *Alive at 5 years, born from 2002 onwards and no missing data (N=19,316)* | | |  |  |
| ED presentations 0-4 years (aIRR) |  |  |  | 0.20 |
|  | Same region | 0.92 (0.87, 0.97) | Ref |  |
|  | Different region | 0.86 (0.79, 0.94) | Ref |  |
| P-value is for the interaction between maternal grandmaternal health and whether she and the child lived in the same Indigenous region at the time the child was born. OR: odds ratio. IRR: incidence rate ratio. Adjusted estimates were obtained from models that included child sex, year of birth, and components of the child's environment: remoteness, socioeconomic disadvantage, maternal age, and maternal smoking during pregnancy. 26,554 children had a maternal grandmother alive when they were born and were alive on their fifth birthday. For 4680 children, the Indigenous region of their grandmother could not be determined and for 51 further children, their own Indigenous region at birth could not be determined. 21,823 remained for this analysis. | | | | |

**Supplementary Table 9: Associations between child health outcomes and health service use and maternal grandmaternal health by number of grandchildren**

|  |  |  | **Maternal grandmother** |  |  |  |  | **Maternal grandfather** |  |  |  |  | **Paternal grandmother** |  |  |  |  | **Paternal grandfather** |  |  |  |
| --- | --- | --- | --- | --- | --- | --- | --- | --- | --- | --- | --- | --- | --- | --- | --- | --- | --- | --- | --- | --- | --- |
| **Child health** | **Number of grandchildren** | **Healthy** | **Unhealthy** | **Deceased** | **P** |  | **Healthy** | **Unhealthy** | **Deceased** | **P** |  | **Healthy** | **Unhealthy** | **Deceased** | **P** |  | **Healthy** | **Unhealthy** | **Deceased** | **P** |  |
| *Alive at 5 years with no missing data* |  |  | N=28,645 |  |  |  |  | N=20,044 |  |  |  |  | N=18,937 |  |  |  |  | N=14,272 |  |  |  |
| Total hospital bed days (aIRR)* |  |  |  |  | 0.02 |  |  |  |  | 0.01 |  |  |  |  | 0.04 |  |  |  |  | 0.21 |  |
|  | 1 | 0.62 (0.52, 0.73) | 0.89 (0.70, 1.12) | Ref |  |  | 0.78 (0.66, 0.92) | 1.21 (0.88, 1.67) | Ref |  |  | 0.73 (0.57, 0.94) | 1.01 (0.72, 1.40) | Ref |  |  | 0.65 (0.49, 0.86) | 0.78 (0.56, 1.08) | Ref |  |  |
|  | 10 | 0.77 (0.68, 0.87) | 0.95 (0.81, 1.13) | Ref |  |  | 0.89 (0.77, 1.02) | 0.89 (0.72, 1.09) | Ref |  |  | 0.69 (0.58, 0.82) | 0.75 (0.60, 0.94) | Ref |  |  | 0.77 (0.63, 0.94) | 0.77 (0.60, 0.98) | Ref |  |  |
|  |  |  |  |  |  |  |  |  |  |  |  |  |  |  |  |  |  |  |  |  |  |
| Potentially avoidable hospital admissions 0-4 years (aIRR) | |  |  |  | 0.02 |  |  |  |  | 0.04 |  |  |  |  | 0.59 |  |  |  |  | 0.03 |  |
|  | 1 | 0.72 (0.63, 0.82) | 0.93 (0.79, 1.09) | Ref |  |  | 0.79 (0.70, 0.88) | 1.00 (0.82, 1.22) | Ref |  |  | 0.76 (0.65, 0.89) | 0.91 (0.72, 1.14) | Ref |  |  | 0.70 (0.57, 0.86) | 0.85 (0.63, 1.15) | Ref |  |  |
|  | 10 | 0.84 (0.77, 0.92) | 1.01 (0.90, 1.13) | Ref |  |  | 0.87 (0.79, 0.95) | 0.93 (0.81, 1.07) | Ref |  |  | 0.77 (0.69, 0.86) | 0.85 (0.73, 0.99) | Ref |  |  | 0.87 (0.77, 0.98) | 0.87 (0.72, 1.05) | Ref |  |  |
|  |  |  |  |  |  |  |  |  |  |  |  |  |  |  |  |  |  |  |  |  |  |
| Any admission for unintentional injury (aOR) | |  |  |  | 0.35 |  |  |  |  | 0.75 |  |  |  |  | 0.05 |  |  |  |  | 0.07 |  |
|  | 1 | 0.79 (0.63, 0.99) | 0.97 (0.72, 1.32) | Ref |  |  | 0.99 (0.81, 1.21) | 1.11 (0.79, 1.55) | Ref |  |  | 0.72 (0.56, 0.92) | 1.02 (0.72, 1.44) | Ref |  |  | 0.84 (0.66, 1.05) | 1.35 (0.93, 1.97) | Ref |  |  |
|  | 10 | 0.92 (0.79, 1.06) | 1.09 (0.90, 1.32) | Ref |  |  | 0.97 (0.84, 1.11) | 0.99 (0.78, 1.24) | Ref |  |  | 0.87 (0.73, 1.02) | 0.98 (0.77, 1.24) | Ref |  |  | 0.81 (0.69, 0.95) | 0.91 (0.69, 1.20) | Ref |  |  |
|  |  |  |  |  |  |  |  |  |  |  |  |  |  |  |  |  |  |  |  |  |  |
| *Alive at 5 years, born from 2002 onwards and no missing data* | |  | N=24,998 |  |  |  | N=17,545 |  |  |  |  | N=16,545 |  |  |  |  | N=12,473 |  |  |  |  |
| ED presentations 0-4 years (aIRR) |  |  |  |  | 0.18 |  |  |  |  | 0.03 |  |  |  |  | 0.58 |  |  |  |  | 0.14 |  |
|  | 1 | 0.90 (0.83, 0.98) | 0.96 (0.87, 1.05) | Ref |  |  | 0.94 (0.87, 1.01) | 1.02 (0.92, 1.14) | Ref |  |  | 0.87 (0.78, 0.96) | 0.96 (0.85, 1.09) | Ref |  |  | 0.85 (0.73, 0.98) | 0.90 (0.77, 1.07) | Ref |  |  |
|  | 10 | 0.93 (0.88, 0.99) | 1.04 (0.97, 1.11) | Ref |  |  | 0.95 (0.90, 1.01) | 0.91 (0.84, 0.99) | Ref |  |  | 0.91 (0.85, 0.98) | 0.99 (0.92, 1.08) | Ref |  |  | 0.95 (0.88, 1.01) | 0.96 (0.87, 1.05) | Ref |  |  |
| P for interaction between grandparental health and number of grandchildren. aOR: adjusted odds ratio with 95% confidence interval. aIRR: adjusted incidence rate ratio with 95% confidence interval. Adjusted estimates were obtained from models that included child sex, year of birth, and components of the child's environment: remoteness, socioeconomic disadvantage, maternal age, and maternal smoking during pregnancy. 30 children were excluded because of incomplete covariate data leaving 28,645 children contributing to the first model. | | | | | | | | | | | | | | | | | | | | | |

**Supplementary Table 10: Associations between child health outcomes and health service use and grandparental health for children whose grandparents had at least one health record in the 10 years before their birth**

|  |  | **Maternal grandmother** | | | |  |  | **Maternal grandfather** | | |  |  | **Paternal grandmother** | | |  |  | **Paternal grandfather** | | |  |
| --- | --- | --- | --- | --- | --- | --- | --- | --- | --- | --- | --- | --- | --- | --- | --- | --- | --- | --- | --- | --- | --- |
| **Child health** | **Adjustment** | **Healthy** | **Unhealthy** | **Deceased** | | **p** |  | **Healthy** | **Unhealthy** | **Deceased** | **p** |  | **Healthy** | **Unhealthy** | **Deceased** | **p** |  | **Healthy** | **Unhealthy** | **Deceased** | **p** |
| *All births with no missing data* |  |  | N=27,628 | |  |  |  |  | N=18,838 |  |  |  |  | N=17,755 |  |  |  |  | N=13,039 |  |  |
| Stillbirth (OR) |  |  |  | |  |  |  |  |  |  |  |  |  |  |  |  |  |  |  |  |  |
|  | YOB and sex | 0.78 (0.53, 1.15) | 1.35 (0.83, 2.20) | | Ref | 0.03 |  | 0.68 (0.48, 0.98) | 0.89 (0.49, 1.62) | Ref | 0.14 |  | 0.63 (0.38, 1.06) | 0.99 (0.50, 1.99) | Ref | 0.14 |  | 0.91 (0.55, 1.49) | 1.30 (0.59, 2.88) | Ref | 0.64 |
|  | Fully adjusted | 0.85 (0.57, 1.25) | 1.37 (0.84, 2.25) | | Ref | 0.06 |  | 0.72 (0.50, 1.03) | 0.90 (0.49, 1.64) | Ref | 0.22 |  | 0.70 (0.42, 1.16) | 1.06 (0.53, 2.12) | Ref | 0.22 |  | 1.00 (0.60, 1.65) | 1.36 (0.61, 3.04) | Ref | 0.73 |
|  |  |  |  | |  |  |  |  |  |  |  |  |  |  |  |  |  |  |  |  |  |
| *Live births with no missing data* |  |  | N=27,297 | |  |  |  |  | N=18,617 |  |  |  |  | N=17,603 |  |  |  |  | N=12,926 |  |  |
| Death before age 5 (OR) |  |  |  | |  |  |  |  |  |  |  |  |  |  |  |  |  |  |  |  |  |
|  | YOB and sex | 0.61 (0.43, 0.86) | 0.98 (0.61, 1.58) | | Ref | 0.01 |  | 0.60 (0.42, 0.87) | 1.00 (0.54, 1.85) | Ref | 0.02 |  | 0.59 (0.37, 0.94) | 0.48 (0.22, 1.09) | Ref | 0.15 |  | 0.84 (0.51, 1.37) | 0.50 (0.14, 1.87) | Ref | 0.45 |
|  | Fully adjusted | 0.67 (0.47, 0.96) | 1.02 (0.63, 1.63) | | Ref | 0.04 |  | 0.69 (0.46, 1.02) | 1.05 (0.57, 1.97) | Ref | 0.11 |  | 0.67 (0.40, 1.11) | 0.51 (0.22, 1.17) | Ref | 0.24 |  | 1.04 (0.63, 1.71) | 0.59 (0.16, 2.16) | Ref | 0.53 |
|  |  |  |  | |  |  |  |  |  |  |  |  |  |  |  |  |  |  |  |  |  |
| *Alive at 5 years with no missing data* | |  | N=26,954 | |  |  |  |  | N=18,408 |  |  |  |  | N=17,451 |  |  |  |  | N=12,818 |  |  |
| Total hospital bed days (IRR)* |  |  |  | |  |  |  |  |  |  |  |  |  |  |  |  |  |  |  |  |  |
|  | YOB and sex | 0.65 (0.58, 0.73) | 0.96 (0.81, 1.13) | | Ref | <0.001 |  | 0.79 (0.69, 0.91) | 1.03 (0.81, 1.31) | Ref | 0.002 |  | 0.70 (0.58, 0.83) | 0.82 (0.65, 1.04) | Ref | <0.001 |  | 0.69 (0.57, 0.83) | 0.81 (0.62, 1.07) | Ref | 0.011 |
|  | Fully adjusted | 0.69 (0.61, 0.78) | 0.93 (0.79, 1.09) | | Ref | <0.001 |  | 0.80 (0.70, 0.91) | 0.94 (0.76, 1.15) | Ref | 0.003 |  | 0.67 (0.56, 0.80) | 0.78 (0.62, 0.98) | Ref | <0.001 |  | 0.69 (0.57, 0.83) | 0.77 (0.61, 0.97) | Ref | 0.012 |
|  |  |  |  | |  |  |  |  |  |  |  |  |  |  |  |  |  |  |  |  |  |
| Potentially avoidable hospital admissions 0-4 years (IRR) | | |  | |  |  |  |  |  |  |  |  |  |  |  |  |  |  |  |  |  |
|  | YOB and sex | 0.76 (0.69, 0.83) | 1.00 (0.89, 1.12) | | Ref | <0.001 |  | 0.82 (0.75, 0.89) | 1.01 (0.87, 1.17) | Ref | <0.001 |  | 0.76 (0.68, 0.84) | 0.88 (0.76, 1.02) | Ref | <0.001 |  | 0.79 (0.70, 0.89) | 0.88 (0.73, 1.05) | Ref | 0.005 |
|  | Fully adjusted | 0.77 (0.71, 0.85) | 0.98 (0.87, 1.10) | | Ref | <0.001 |  | 0.80 (0.73, 0.87) | 0.94 (0.81, 1.08) | Ref | <0.001 |  | 0.75 (0.67, 0.83) | 0.85 (0.73, 0.99) | Ref | <0.001 |  | 0.77 (0.68, 0.88) | 0.86 (0.71, 1.03) | Ref | 0.006 |
|  |  |  |  | |  |  |  |  |  |  |  |  |  |  |  |  |  |  |  |  |  |
| Any admission for unintentional injury (OR) | |  |  | |  |  |  |  |  |  |  |  |  |  |  |  |  |  |  |  |  |
|  | YOB and sex | 0.93 (0.81, 1.07) | 1.13 (0.93, 1.36) | | Ref | 0.03 |  | 0.98 (0.86, 1.13) | 1.04 (0.83, 1.30) | Ref | 0.85 |  | 0.88 (0.75, 1.03) | 1.05 (0.83, 1.31) | Ref | 0.07 |  | 0.84 (0.72, 0.97) | 1.08 (0.84, 1.40) | Ref | 0.02 |
|  | Fully adjusted | 0.89 (0.77, 1.02) | 1.07 (0.89, 1.30) | | Ref | 0.02 |  | 0.94 (0.81, 1.07) | 1.00 (0.80, 1.26) | Ref | 0.54 |  | 0.83 (0.70, 0.97) | 0.98 (0.78, 1.24) | Ref | 0.02 |  | 0.79 (0.67, 0.92) | 1.01 (0.78, 1.30) | Ref | 0.006 |
|  |  |  |  | |  |  |  |  |  |  |  |  |  |  |  |  |  |  |  |  |  |
| Any unavoidable admissions (OR) |  |  |  | |  |  |  |  |  |  |  |  |  |  |  |  |  |  |  |  |  |
|  | YOB and sex | 0.99 (0.77, 1.28) | 1.20 (0.87, 1.67) | | Ref | 0.34 |  | 0.95 (0.74, 1.21) | 1.18 (0.79, 1.75) | Ref | 0.49 |  | 0.89 (0.67, 1.18) | 0.96 (0.64, 1.43) | Ref | 0.67 |  | 0.79 (0.62, 1.02) | 0.66 (0.40, 1.09) | Ref | 0.12 |
|  | Fully adjusted | 0.98 (0.77, 1.26) | 1.19 (0.86, 1.65) | | Ref | 0.36 |  | 0.92 (0.72, 1.18) | 1.16 (0.78, 1.73) | Ref | 0.41 |  | 0.89 (0.66, 1.18) | 0.95 (0.64, 1.42) | Ref | 0.68 |  | 0.80 (0.62, 1.04) | 0.67 (0.41, 1.11) | Ref | 0.14 |
|  |  |  |  | |  |  |  |  |  |  |  |  |  |  |  |  |  |  |  |  |  |
| *Alive at 5 years, born from 2002 onwards and no missing data* | | | N=23,702 | |  |  |  |  | N=16,219 |  |  |  |  | N=15,394 |  |  |  |  | N=11,293 |  |  |
| ED presentations 0-4 years (IRR) |  |  |  | |  |  |  |  |  |  |  |  |  |  |  |  |  |  |  |  |  |
|  | YOB and sex | 0.92 (0.87, 0.97) | 1.04 (0.97, 1.11) | | Ref | <0.001 |  | 0.99 (0.93, 1.05) | 1.01 (0.93, 1.09) | Ref | 0.76 |  | 0.93 (0.86, 0.99) | 1.02 (0.94, 1.11) | Ref | <0.001 |  | 0.94 (0.87, 1.01) | 0.96 (0.87, 1.05) | Ref | 0.28 |
|  | Fully adjusted | 0.91 (0.86, 0.95) | 1.01 (0.95, 1.08) | | Ref | <0.001 |  | 0.93 (0.88, 0.98) | 0.93 (0.86, 1.00) | Ref | 0.03 |  | 0.89 (0.83, 0.95) | 0.98 (0.90, 1.07) | Ref | <0.001 |  | 0.89 (0.82, 0.96) | 0.93 (0.84, 1.03) | Ref | 0.05 |
| p: P-value. YOB: Year of birth. OR: odds ratio with 95% confidence interval. IRR: incidence rate ratio with 95% confidence interval. YOB: year of birth. Fully adjusted estimates were obtained from models that included child sex, year of birth, and components of the child's environment: remoteness, socioeconomic disadvantage, maternal age, and maternal smoking during pregnancy. 28 children were excluded because of incomplete covariate data leaving 27,628 children contributing to the first model. Each estimate is from a separate model, with the health of only one grandparent included in each model. | | | | | | | | | | | | | | | | | | | | | |

**Supplementary Table 11: Associations between child health outcomes and health service use and the number of Elixhauser comorbidities experienced by grandparents who were alive at the time of the child’s birth**

|  | **Maternal grandmother comorbidities** | | | |  | **Maternal grandfather comorbidities** | | | |  | **Paternal grandmother comorbidities** | | | |  | **Paternal grandfather comorbidities** | | | |
| --- | --- | --- | --- | --- | --- | --- | --- | --- | --- | --- | --- | --- | --- | --- | --- | --- | --- | --- | --- |
| **Child health** | **0 n (%)** | **1 n (%)** | **2 n (%)** | **3 or more n (%)** |  | **0 n (%)** | **1 n (%)** | **2 n (%)** | **3 or more n (%)** |  | **0 n (%)** | **1 n (%)** | **2 n (%)** | **3 or more n (%)** |  | **0 n (%)** | **1 n (%)** | **2 n (%)** | **3 or more n (%)** |
| *All births* |  |  | N=27,217 |  |  |  |  | N=17,858 |  |  |  |  |  | N=17,747 |  |  |  | N=12,456 |  |
| Stillbirth |  |  |  |  |  |  |  |  |  |  |  |  |  |  |  |  |  |  |  |
| Yes | 268 (1) | 22 (1) | 19 (2) | 21 (2) |  | 175 (1) | 9 (1) | 6 (1) | 9 (2) |  | 131 (1) | 6 (1) | n.p. | 12 (2) |  | 97 (1) | 5 (1) | n.p. | n.p. |
| No | 23102 (99) | 1759 (99) | 982 (98) | 1044 (98) |  | 15813 (99) | 782 (99) | 478 (99) | 586 (98) |  | 15300 (99) | 1021 (99) | n.p. | 676 (98) |  | 11034 (99) | 567 (99) | n.p. | n.p. |
|  |  |  |  |  |  |  |  |  |  |  |  |  |  |  |  |  |  |  |  |
| *Live births* |  |  | N=26,887 |  |  |  |  | N=17,659 |  |  |  |  |  | N=17,594 |  |  |  | N=12,345 |  |
| Death before age 5 |  |  |  |  |  |  |  |  |  |  |  |  |  |  |  |  |  |  |  |
| Yes | 269 (1) | 27 (2) | 23 (2) | 14 (1) |  | 164 (1) | 7 (1) | 7 (1) | 11 (2) |  | 125 (1) | 11 (1) | n.p. | n.p. |  | 88 (1) | 5 (1) | n.p. | n.p. |
| No | 22833 (99) | 1732 (98) | 959 (98) | 1030 (99) |  | 15649 (99) | 775 (99) | 471 (99) | 575 (98) |  | 15175 (99) | 1010 (99) | n.p. | n.p. |  | 10946 (99) | 562 (99) | n.p. | n.p. |
|  |  |  |  |  |  |  |  |  |  |  |  |  |  |  |  |  |  |  |  |
| *Alive at 5 years* |  |  | N=26,554 |  |  |  |  | N=17,470 |  |  |  |  |  | N=17,449 |  |  |  | N=12,248 |  |
| Total hospital bed days (excluding birth admission) | | |  |  |  |  |  |  |  |  |  |  |  |  |  |  |  |  |  |
| Median (IQR) | 1 (0-4) | 1 (0-5) | 2 (0-5) | 2 (0-6) |  | 1 (0-3) | 1 (0-4) | 1 (0-4) | 1 (0-4) |  | 1 (0-4) | 1 (0-4) | 1 (0-4) | 1 (0-4) |  | 1 (0-3) | 1 (0-4) | 1 (0-3) | 1 (0-4) |
| 0 | 9303 (41) | 617 (36) | 314 (33) | 320 (31) |  | 6698 (43) | 293 (38) | 196 (42) | 221 (38) |  | 6235 (41) | 387 (38) | 213 (36) | 248 (37) |  | 4729 (43) | 225 (40) | 132 (41) | 168 (40) |
| 1-3 | 7444 (33) | 542 (31) | 292 (30) | 328 (32) |  | 5221 (33) | 275 (35) | 150 (32) | 187 (33) |  | 5123 (34) | 345 (34) | 204 (34) | 226 (34) |  | 3768 (34) | 191 (34) | 110 (34) | 136 (32) |
| 4-6 | 2555 (11) | 218 (13) | 154 (16) | 132 (13) |  | 1757 (11) | 83 (11) | 51 (11) | 72 (13) |  | 1698 (11) | 104 (10) | 76 (13) | 86 (13) |  | 1146 (10) | 72 (13) | 31 (10) | 51 (12) |
| 7+ | 3531 (15) | 355 (20) | 199 (21) | 250 (24) |  | 1973 (13) | 124 (16) | 74 (16) | 95 (17) |  | 2119 (14) | 174 (17) | 99 (17) | 112 (17) |  | 1303 (12) | 74 (13) | 46 (14) | 66 (16) |
| Total potentially avoidable hospital admissions 0-4 years | | |  |  |  |  |  |  |  |  |  |  |  |  |  |  |  |  |  |
| Median (IQR) | 0 (0-1) | 0 (0-1) | 0 (0-1) | 0 (0-1) |  | 0 (0-1) | 0 (0-1) | 0 (0-1) | 0 (0-1) |  | 0 (0-1) | 0 (0-1) | 0 (0-1) | 0 (0-1) |  | 0 (0-1) | 0 (0-1) | 0 (0-1) | 0 (0-1) |
| 0 | 14678 (64) | 992 (57) | 516 (54) | 547 (53) |  | 10509 (67) | 505 (65) | 294 (62) | 357 (62) |  | 9922 (65) | 636 (63) | 366 (62) | 420 (62) |  | 7469 (68) | 377 (67) | 207 (65) | 274 (65) |
| 1 | 4803 (21) | 395 (23) | 225 (23) | 247 (24) |  | 3222 (21) | 154 (20) | 102 (22) | 133 (23) |  | 3180 (21) | 218 (22) | 134 (23) | 149 (22) |  | 2185 (20) | 125 (22) | 66 (21) | 91 (22) |
| 2+ | 3352 (15) | 345 (20) | 218 (23) | 236 (23) |  | 1918 (12) | 116 (15) | 75 (16) | 85 (15) |  | 2073 (14) | 156 (15) | 92 (16) | 103 (15) |  | 1292 (12) | 60 (11) | 46 (14) | 56 (13) |
| Any admission for unintentional injury | |  |  |  |  |  |  |  |  |  |  |  |  |  |  |  |  |  |  |
| No | 20312 (89) | 1524 (88) | 833 (87) | 890 (86) |  | 13981 (89) | 669 (86) | 415 (88) | 512 (89) |  | 13486 (89) | 879 (87) | 534 (90) | 563 (84) |  | 9784 (89) | 496 (88) | 276 (87) | 364 (86) |
| Yes | 2521 (11) | 208 (12) | 126 (13) | 140 (14) |  | 1668 (11) | 106 (14) | 56 (12) | 63 (11) |  | 1689 (11) | 131 (13) | 58 (10) | 109 (16) |  | 1162 (11) | 66 (12) | 43 (13) | 57 (14) |
| Any unavoidable admissions |  |  |  |  |  |  |  |  |  |  |  |  |  |  |  |  |  |  |  |
| No | 22080 (97) | 1664 (96) | 923 (96) | 986 (96) |  | 15161 (97) | 740 (95) | 456 (97) | 549 (95) |  | 14690 (97) | 965 (96) | 570 (96) | 648 (96) |  | 10598 (97) | 544 (97) | 311 (97) | 409 (97) |
| Yes | 753 (3) | 68 (4) | 36 (4) | 44 (4) |  | 488 (3) | 35 (5) | 15 (3) | 26 (5) |  | 485 (3) | 45 (4) | 22 (4) | 24 (4) |  | 348 (3) | 18 (3) | 8 (3) | 12 (3) |
|  |  |  |  |  |  |  |  |  |  |  |  |  |  |  |  |  |  |  |  |
| *Alive at 5 years and born from 2002 onwards* | |  | N=23,182 |  |  |  |  | N=15,307 |  |  |  |  |  | N=15,255 |  |  |  | N=10,724 |  |
| Total ED presentations 0-4 years |  |  |  |  |  |  |  |  |  |  |  |  |  |  |  |  |  |  |  |
| Median (IQR) | 5 (2-10) | 6 (3-12) | 7 (3-13) | 6 (3-13) |  | 5 (2-10) | 6 (2-10) | 5 (2-10) | 6 (3-10) |  | 5 (2-10) | 6 (3-11) | 6 (3-12) | 6 (2-11) |  | 5 (2-9) | 5 (2-11) | 6 (3-10) | 5 (2-9) |
| 0 | 1584 (8) | 107 (7) | 53 (6) | 67 (7) |  | 1142 (8) | 54 (8) | 30 (7) | 36 (7) |  | 919 (7) | 51 (6) | 19 (4) | 36 (6) |  | 713 (7) | 34 (7) | 18 (6) | 17 (5) |
| 1-4 | 7187 (36) | 458 (30) | 240 (29) | 272 (30) |  | 5181 (38) | 233 (34) | 157 (38) | 175 (34) |  | 4890 (37) | 315 (35) | 178 (34) | 200 (34) |  | 3706 (39) | 183 (38) | 100 (35) | 138 (37) |
| 5+ | 11145 (56) | 947 (63) | 542 (65) | 580 (63) |  | 7360 (54) | 406 (59) | 230 (55) | 303 (59) |  | 7430 (56) | 533 (59) | 328 (62) | 356 (60) |  | 5165 (54) | 266 (55) | 166 (58) | 218 (58) |
| ED: Emergency Department. IQR: Interquartile range. n.p.=counts are not publishable because of privacy concerns as they are less than 5 or could lead to calculation of a small count. | | | | | | | | | | | | | | | | | | | |
